# Arylthiazoline Unit as a Ferric Ion Chelating Moiety in Siderophores: The Coordination Chemistry of Anguibactin and Its Analogs

**DOI:** 10.1021/acs.inorgchem.6c03994

**Published:** 2026-09-01

**Authors:** Klaudia Szczerba, Andrzej Mular, Karolina Piasta, Karolina Kamińska, Oscar Palacios, Mercè Capdevila, Elżbieta Wojaczyńska, Elżbieta Gumienna-Kontecka

**Affiliations:** † Faculty of Chemistry, University of Wrocław, F. Joliot-Curie 14, 50-383 Wrocław, Poland; ‡ Faculty of Chemistry, Wrocław University of Science and Technology, Wybrzeże Wyspiańskiego 27, 50-370 Wrocław, Poland; § Departament de Química, Universitat Autònoma de Barcelona, 08193 Cerdanyola del Vallès, Spain

## Abstract

The 2-(2-hydroxyphenyl)- and 2-(2,3-dihydroxyphenyl)­thiazoline motifs appear repeatedly across microbial siderophores, yet the thermodynamic properties of their Fe­(III) complexes remain fragmentarily described. Herein we present the Fe­(III) coordination chemistry of anguibactin (**Ang**), the siderophore of *Vibrio anguillarum*, together with four designed analogs (**L1–L4**) that pair a phenol- or catechol-thiazoline core with an auxiliary donor group, either carboxylate or hydroxamate, analyzing the contribution of each functional group against a common scaffold. Using physicochemical analyses in MeOH/H_2_O (80:20, w/w), we determined the protonation equilibria, complex stoichiometries, pH-dependent speciation, and Fe­(III) affinities. At physiological pH all ligands form bis-ligand complexes in which iron is bound through the phenolate–thiazoline unit and the auxiliary donor rather than a catecholate sphere. Replacing the carboxylate by a hydroxamate unit substantially increases Fe­(III) affinity. Of importance, **Ang** retains the highest affinity of the series (pFe = 28.2), exceeding that of closely related desferrithiocin and being close to other efficient siderophores, like tris­(hydroxamate) ferrioxamines or pyoverdine, a peptide-based siderophore comprising dihydroxyquinoline, hydroxamate, and catecholate motifs. As **Ang** has been proposed as a thermally stable counterpart to acinetobactin – a siderophore from the multidrug-resistant pathogen *Acinetobacter baumannii*, the structure–property relationships can support the future design of arylthiazoline-based “Trojan horse” siderophore–antibiotic conjugates.

## Graphical abstract

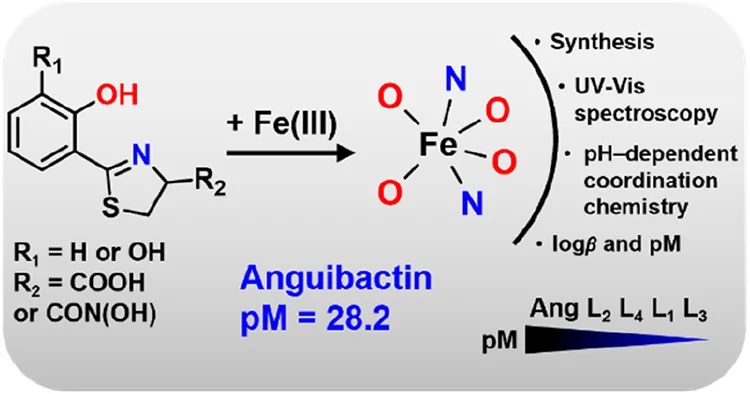

## Introduction

The low bioavailability of iron, which at neutral pH is largely immobilized as insoluble Fe­(III) oxides and hydroxides, has driven microorganisms to evolve low-molecular-weight, high affinity Fe­(III)-binding scavengers, siderophores, that are secreted into the extracellular medium to chelate Fe­(III) ions.^1^ The arylthiazoline scaffold, particularly the 2-(2-hydroxyphenyl)-thiazoline (phenol-thiazoline) and 2-(2,3-dihydroxyphenyl)-thiazoline (catechol-thiazoline) motifs, is found throughout microbial siderophores, as well as in their biosynthetic shunt and degradation products, and in many other natural compounds.^2–5^ Beyond metal acquisition, compounds built on this fragment exhibit a remarkably broad spectrum of biological activities, spanning antitumor, antibiotic, anti-inflammatory, and antiviral effects, which makes them attractive scaffolds in contemporary drug discovery.^6–8^ It is worth distinguishing between the aromatic thiazole, the thiazoline (4,5-dihydrothiazole) and the fully saturated thiazolidine (tetrahydrothiazole) rings encountered across the siderophore family. This change in ring saturation modulates the electronic structure of the chelating fragment, the basicity of the ring nitrogen, and the geometric preferences upon coordination, and is therefore an important structural variable when comparing closely related natural chelators.^9–12^

In almost all naturally occurring arylthiazoline-based chelators, the phenol/catechol–thiazoline unit is by itself insufficient to satisfy the hexacoordinate preference of Fe­(III). Instead, the same molecule carries one or more auxiliary donor groups that raise its denticity to tetra- through hexadentate. Where the donor set remains incomplete (most notably in tetradentate chelators such as desferrithiocin) octahedral coordination of the metal ion is achieved by binding two ligand molecules, giving a 1:2 (metal/ligand) complex.^13^ The accessory functions are remarkably varied: carboxylates, as in aeruginoic acid (AA), dihydroaeruginoic acid (DHA), desferrithiocin and pyochelin, hydroxamates and imidazole ring as in anguibactin (**Ang**), and further thiazolidine rings, as exemplified by pyochelin, yersiniabactin, micacocidin, piscibactin, and ulbactins.^2–4^ AA and DHA (Figure 1) are degradation products of pyochelin, siderophore produced by *Pseudomonas aeruginosa*. AA and DHA are the simplest phenol–thiazole and phenol–thiazoline chelators, respectively, each extended by a single carboxylate unit. Recent work has shown that under moderate iron deficiency, when only pyochelin is produced and not pyoverdine (the second, higher-affinity siderophore of *P. aeruginosa*), AA and DHA enhance iron uptake by inducing femA expression and facilitating iron transport through the ferri-mycobactin outer membrane transporter FemA. It provided new insights into the pathogen’s strategies for iron homeostasis using siderophore metabolites.^14,15^

**Figure 1:**
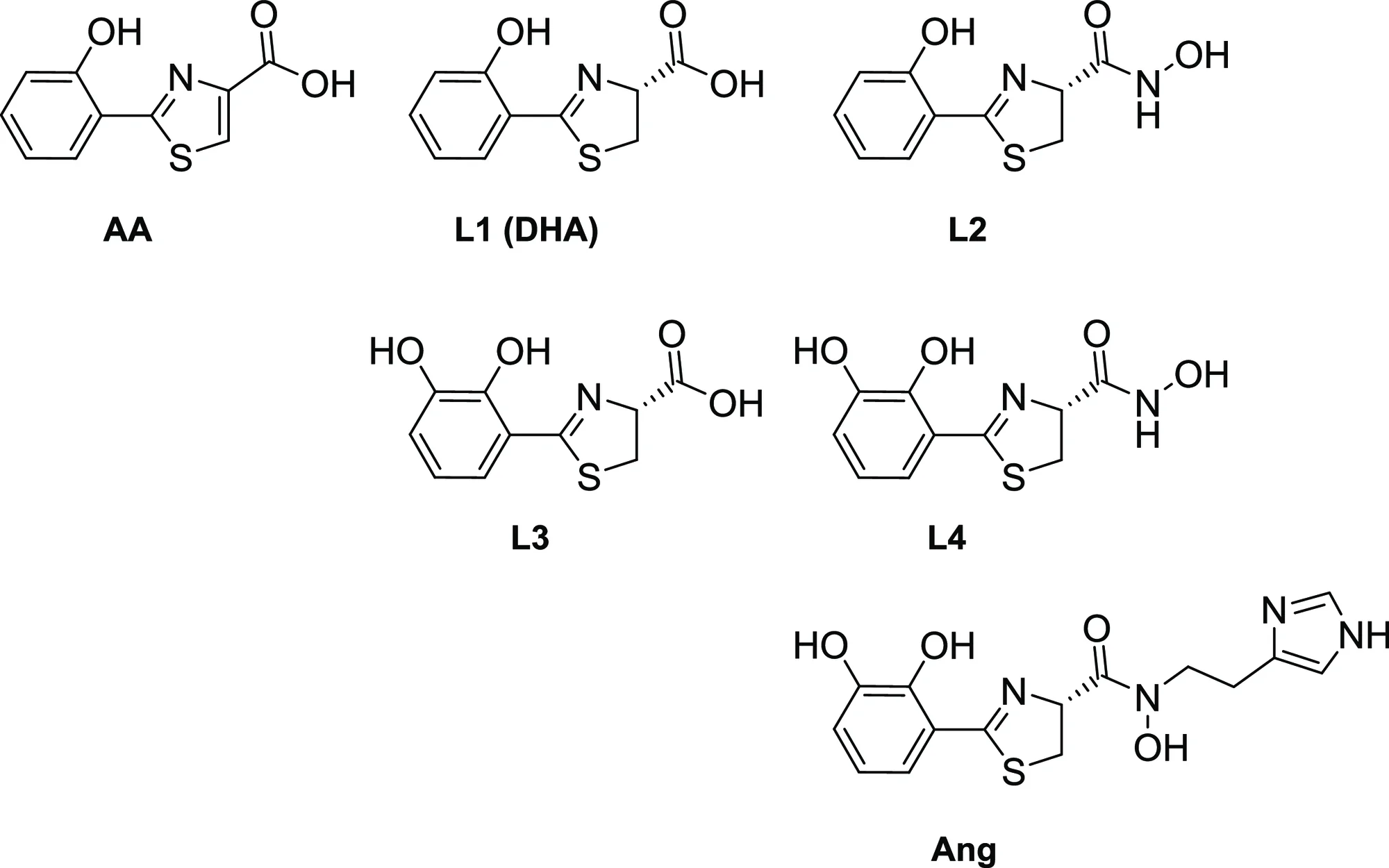
Structures of aeruginoic acid (AA), dihydroaeruginoic acid (DHA = **L1**), **L2**, **L3**, **L4** and anguibactin (**Ang**).

**Ang**, a siderophore of fish pathogen *Vibrio anguillarum*, comprises an *N*-hydroxy-*N*-acyl-histamine-derived hydroxamate unit with a 2-(2,3-dihydroxyphenyl)-thiazoline motif (Figure 1). Early characterization of an Fe­(III)–**Ang** complex established a 1:1 metal-to-ligand stoichiometry,^16,17^ and the crystal structure of a Ga­(III) (a biomimetic of iron) 2:2 complex revealed a coordination sphere built from the catecholate oxygen, thiazoline nitrogen, deprotonated hydroxamate oxygen and imidazole nitrogen of each ligand.^18^ Based on fluorescent titration Job Plot, a 1:2 stoichiometry was subsequently proposed for Fe­(III)–**Ang**.^19,20^ Studies of **Ang** coordination chemistry extend to the closely related acinetobactin siderophore, produced by the multidrug-resistant opportunistic pathogen *Acinetobacter baumannii*, a common cause of fatal hospital-acquired infections.^21^ Its oxazoline-containing form, preacinetobactin, is the direct structural analog of **Ang**, differing only in the ring heteroatom (O in place of S). Notably, **Ang** could serve as a thermally stable counterpart to the labile preacinetobactin, given the similarity of their cellular uptake through the Bau transport system.^19^ Acinetobactin was proposed before as a drug carrier in *A. baumanii* infections, yet the main drawback of this compound was a thermal instability of the oxazoline ring.^3^

Given that **Ang** bears four potential metal-coordinating moieties (the catecholate and hydroxamate groups and the thiazoline and imidazole rings), the coordination sphere adopted by its complex at any given pH remains ambiguous and difficult to predict a priori. The influence of the hydroxamate group on Fe­(III) acquisition in analogous mixed-donor siderophores has been a matter of debate.^20,22,23^ In the oxazoline-based siderophores preacinetobactin and prepseudomonine, the hydroxamate group has a role that extends beyond Fe­(III) coordination. Its *N*-hydroxamate oxygen acts as an intramolecular nucleophile that drives the nonenzymatic isomerization of the 2-(2,3-dihydroxyphenyl)­oxazoline core to the corresponding isoxazolidinone.^20,22^ **Ang**, the thiazoline counterpart of this series, does not undergo an analogous pH-dependent isomerization.^19^ Consequently, the influence of the hydroxamate group in the arylthiazoline series is expected to be primarily coordinative and thermodynamic, rather than coupled to a pH-triggered structural rearrangement as in the oxazoline analogs.

Beyond their native role in microbial iron acquisition, siderophores are of growing interest as scaffolds for antimicrobial drug design.^24^ Because pathogens depend on siderophore-mediated iron uptake for virulence, their dedicated transport machinery can be hijacked to deliver antibiotics into the cell (the “Trojan horse” strategy exploited by siderophore–antibiotic conjugates).^25^ The viability of such conjugates rests on the metal complex itself: the donor set, protonation state, and stoichiometry adopted by the Fe­(III) complex govern how it is recognized and internalized by the cognate receptor.^26^ A predictive understanding of the pH-dependent coordination chemistry of these ligands is therefore a prerequisite for rational conjugate design, not merely a descriptive detail. Notwithstanding the structural diversity and biological importance of this family, the literature on the thermodynamic stability, speciation and coordination geometry of arylthiazoline-based metal complexes remains surprisingly fragmentary, with most reports providing only incremental data.^3^ Detailed solution-equilibrium and structural studies have been disclosed for only a handful of representatives: pyochelin and its diastereomers,^27,28^ the desferrithiocin family together with its early synthetic analogs,^13,29^ and the synthetic bis­((2-hydroxyphenyl)-thiazoline-carboxamide) (BHPTC) chelators recently developed as desferrithiocin-inspired iron mobilizers.^30^ As a consequence, structure–coordination relationships across the broader arylthiazoline siderophore family are still far from fully understood. Even for well-known members such as yersiniabactin,^31^ piscibactin^32^ or ulbactin F,^33^ the pH-dependent speciation is incompletely characterized, and the situation is similar for **Ang** itself. This uncertainty is further complicated by the structural complexity of these ligands, which typically present several potential chelating groups whose relative participation shifts with pH. We therefore studied **Ang** alongside a series of smaller, designed analogs: phenol-thiazoline and catechol-thiazoline derivatives bearing either a carboxylate or a hydroxamate auxiliary group (**L1–L4**, Figure 1). These analogs were chosen to understand the contribution of each additional chelating motif within an otherwise comparable scaffold, allowing the effect of a single donor group to be assessed against a common structural background. Our aim is to determine how each auxiliary group shapes the physicochemical properties of arylthiazoline chelators, and thereby to build the structure–property basis needed to interpret coordination behavior and speciation of ferric complexes of this class of ligands. The inclusion of **L1** serves a dual purpose: it provides a structurally minimal model of the phenol-thiazoline–carboxylate fragment, and it is itself a biologically relevant Fe­(III) ligand (as described previously).

## Experimental Section

### Synthesis of L1–L4

#### General Considerations

Compounds **L1** and **L3** have been previously reported and were prepared according to published procedures for **L1** ^34^ and **L3**.^19^ To the best of our knowledge, the hydroxamic-acid derivatives **L2** and **L4** are new compounds and are reported here for the first time. **L2** and **L4** were prepared from **L1** and **L3**, respectively, using the general hydroxamic-acid formation methodology reported by Giacomelli et al.^35^ For the previously reported compounds **L1** and **L3**, abbreviated preparation and characterization data are provided below, whereas full experimental and analytical data are given for the new compounds **L2** and **L4**.

For the synthesis commercially available reagents and solvents were purchased and used as received unless otherwise stated. NMR spectra were recorded on a Bruker AVANCE NEO 400 OneBay spectrometer and are provided in the Supporting Information. Chemical shifts are reported in δ (ppm) and referenced to the residual solvent signal as an internal standard. The following abbreviations are used: s = singlet, d = doublet, t = triplet, m = multiplet, and bs = broad singlet. IR spectra were recorded on a Shimadzu IR Prestige-21 spectrometer. ESI mass spectra were measured on a Bruker micrOTOF-II-SF spectrometer. Melting points were determined using a Yanaco micro melting point apparatus.

##### (4*R*)-2-(2-Hydroxyphenyl)-4,5-dihydro-1,3-thiazole-4-carboxylic Acid (L1)

Compound was prepared according to the reported procedure (Scheme 1),^34^ and obtained as a yellow powder (1.69 g, 90%); mp 118.5–120 °C. The spectroscopic data were consistent with the literature data.^34^

**Scheme 1:**
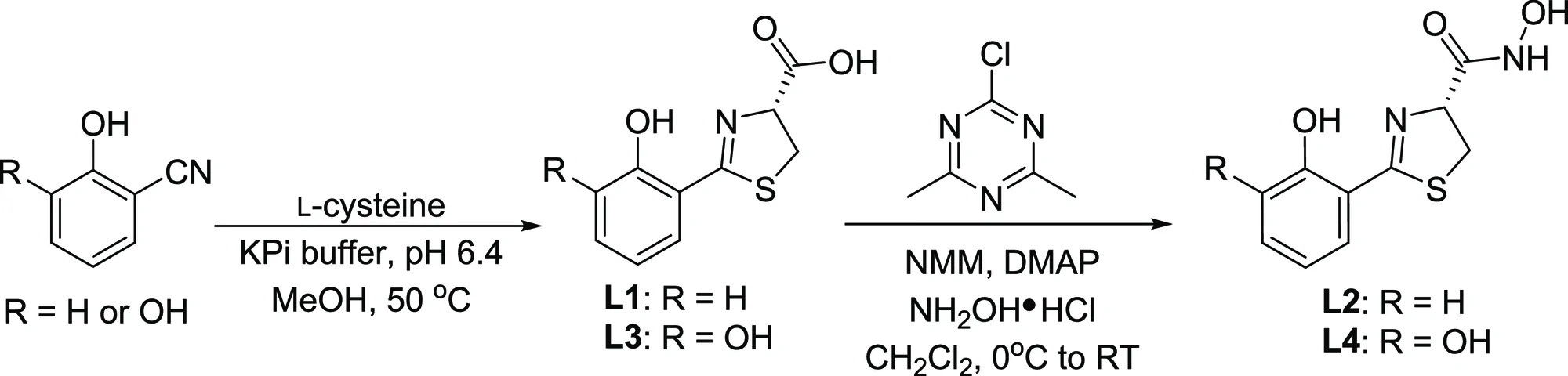
Synthesis of **L1**–**L4**

##### (4*R*)-2-(2,3-Dihydroxyphenyl)-4,5-dihydro-1,3-thiazole-4-carboxylic Acid (L3)

Compound was prepared according to the reported procedure,^19^ and obtained as a yellow powder (1.76 g, 60%); mp 190–191.5 °C. The spectroscopic data were consistent with the literature data.^19^

##### (4*R*)-*N*-Hydroxy-2-(2-hydroxyphenyl)-4,5-dihydro-1,3-thiazole-4-carboxamide (L2) and (4*R*)-*N*-Hydroxy-2-(2,3-dihydroxyphenyl)-4,5-dihydro-1,3-thiazole-4-carboxamide (L4)

Compounds were synthesized according to a reported hydroxamic-acid formation procedure.^35^ Cyanuric chloride (0.129 g, 0.700 mmol) was added at 0 °C to a stirred solution of the corresponding carboxylic acid (**L1**, 0.468 g, 2.10 mmol, or **L3**, 0.502 g, 2.10 mmol), containing *N*-methylmorpholine (NMM; 0.253 mL, 2.31 mmol), 4-dimethylaminopyridine (DMAP; 3.0 mg, 0.021 mmol), and hydroxylamine hydrochloride (0.160 g, 2.31 mmol) in CH_2_Cl_2_ (10 mL). After the addition, the mixture was warmed to room temperature and stirred for 12 h. The mixture was filtered through Celite, and the organic phase was washed with 1 M HCl (3 × 15 mL) and then with brine. The organic layer was dried over Na_2_SO_4_, filtered, and evaporated to afford the final product without additional purification. **L2**: off-white solid; yield 0.18 g (36%); mp 119–122 °C. ^1^H NMR (400 MHz, CD_3_OD) δ 7.49 (dd, *J* = 7.9, 1.5 Hz, 1H), 7.41 (td, *J* = 7.8, 1.6 Hz, 1H), 6.97 (dd, *J* = 7.7 Hz, 1.0 Hz, 1H), 6.93 (td, *J* = 7.6, 1.1 Hz, 1H), 5.22 (t, *J* = 9.2 Hz, 1H), 3.66 (d, *J* = 9.2 Hz, 2H). ^13^C NMR (100 MHz, CD_3_OD) δ 174.4, 167.8, 158.7, 133.2, 130.5, 118.8, 116.6, 116.0, 76.4, 32.2. HRMS (ESI+) *m*/*z*: [M + H]^+^ calcd for C_10_H_11_N_2_O_3_S, 239.0485; found, 239.0695. **L4**: off-white solid; yield 0.32 g (40%); mp 120–121 °C. ^1^H NMR (400 MHz, CD_3_OD) δ 6.98 (dd, *J* = 7.9, 1.5 Hz, 1H), 6.94 (dd, *J* = 7.9, 1.5 Hz, 1H), 6.76 (t, *J* = 7.9 Hz, 1H), 5.20 (t, *J* = 9.2 Hz, 1H), 3.64 (d, *J* = 9.2 Hz, 2H). ^13^C NMR (100 MHz, CD_3_OD) δ 176.3, 169.4, 148.5, 147.0, 122.3, 120.1, 119.9, 117.4, 77.6, 34.4. HRMS (ESI+) *m*/*z*: [M + H]^+^ calcd for C_10_H_11_N_2_O_4_S, 255.0434; found, 255.0834.

### Thermodynamic Solution Studies

#### General Considerations

All reagents and solvents, with the exception of **L1–L4** analogs, used in the thermodynamic studies were purchased from commercial suppliers. The obtained compounds were of analytical grade and were used without further purification. Solutions were prepared using doubly distilled water. Fe­(III) solutions were freshly prepared on the day of measurement by dissolving Fe­(ClO_4_)_3_·*x*H_2_O (Sigma-Aldrich) in 0.01 M HClO_4_ (*caution:* perchlorate salts combined with organic ligands are potentially explosive and should be handled in small quantity and with the necessary precautions).^36^ The iron concentration in solution was determined spectrophotometrically based on a molar extinction coefficient ε = 4.16 × 10^3^ M^–1^ cm^–1^ at 240 nm.^37,38^ All the solution studies were carried out in methanol–water (80:20 w/w) mixture. The ionic strength was adjusted to 0.1 M with NaClO_4_. The p*K*_w_ used in the spectrophotometric and potentiometric calculations was 14.42. The HClO_4_ solutions were titrated against a 0.1 M NaOH solution, while the carbonate-free NaOH solution was standardized by titrations using potassium hydrogen phthalate (KHP, Chempur, >99.5%). Ferrocene (Ventron, >99%) was used for electrochemical measurements.

Electrospray ionization mass spectrometry (ESI-MS) measurements were carried out using a Bruker mass spectrometer equipped with an electrospray ionization (ESI) source and a timsTOF Pro 2 or a Q-TOF compact mass analyzer. The method was applied in the *m*/*z* range of 50–2000, with nitrogen as the drying gas. Temperature was set as 70 °C for **L1–L4** and 180 °C for **Ang**, while the potential difference between the needle and the capillary inlet was 3500 V for **L1–L4** and 4500 V for **Ang**. The final complex concentration in the sample was 0.1 mM, with a metal-to-ligand molar ratio of 1:2 for all ligands and also 1:3 for **L3**, **L4** and **Ang**. The initial pH of the Fe­(III) complex solutions for **L1–L4** was approximately 7. In the case of **Ang**, the solution was measured without the addition of ammonia and after adding a portion of ammonia. Measurements were conducted in the positive ion mode.

pH-Dependent ultraviolet–visible (UV–Vis) spectrophotometric titrations for the ligands and Fe­(III) complexes, were carried out as a function of concentration using a Varian Cary 300 Bio or Cary 50 spectrophotometer. Spectra were recorded in the 200–450 nm range (with 0.5 nm precision) for ligands, and 200–800 nm for the Fe­(III) complexes. Measurements were performed at 25 °C ± 0.1 °C using Hellma quartz cuvettes or Ultra Mini Immersion probe with a 1 cm optical path length. For ligands **L3**, **L4**, and **Ang**, measurements were performed on degassed solutions under anaerobic conditions either in a glovebox or in a sealed potentiometric vessel under an argon atmosphere using a coupled potentiometric-spectrophotometric setup. The pH was monitored using a Beckman 72 pH meter with an accuracy of ±0.001, and the corresponding UV–vis spectrum was subsequently recorded. For titrations of ligands, solutions of *C*_L_ in the range of 3–5 × 10^–5^ M were prepared. For titrations of Fe­(III) complexes, solutions of 0.5–5 × 10^–4^ M complex for **L1**, **L2** and **Ang** (M/L = 1:2) and 0.25–1 × 10^–4^ M (M/L = 1:4) for **L3** and **L4** were prepared. The pH was controlled by adjusting the concentrations of HClO_4_ and NaOH, respectively.

The data were refined using SPECFIT/32 software which adjusts the absorptivity and the stability constants of the species formed at equilibrium.^39,40^ SPECFIT/32 uses factor analysis to reduce the absorbance matrix and to extract the eigenvalues prior to the multiwavelength fit of the reduced data set according to the Marquardt algorithm.^39,40^ Stepwise acid dissociation constants of the ligand were derived from the overall stability constants determined by the program and defined by eqs ([Disp-formula eq1]) and ([Disp-formula eq2]) (charges omitted for clarity).

1 $$H_{n}L⇄H_{n-1}L+H$$

2 $$K_{a,n}=\frac{\left[H_{n-1}L\right]\left[H\right]}{\left[H_{n}L\right]}$$

The stability constants calculated for metal complexes are defined by eqs ([Disp-formula eq3]) and ([Disp-formula eq4])­

3 $$pM+qH+rL⇄M_{p}H_{q}L_{r}$$

4 $$\beta_{M_{p}H_{q}L_{r}}=\frac{\left[M_{p}H_{q}L_{r}\right]}{{\left[M\right]}^{p}{\left[H\right]}^{q}{\left[L\right]}^{r}}$$

In the calculations of complex stability constants, the deprotonation constants of free ligand and the constants related to hydrolytic Fe­(III) species were taken into account: log β_FeH–1_ = −2.56, log β_FeH–2_ = −6.20, log β_FeH–3_ = −11.44, log β_FeH–4_ = −21.88, log β_Fe2H–2_ = −2.74.^41^ The species distribution diagrams were computed with HySS program.^42^ Uncertainties in log β were calculated from the standard deviation (σ). The data were processed using Origin 2026.

Potentiometric titrations were performed using an automatic titration system, Metrohm Titrando 809. Data acquisition was carried out with the Tiamo 2.5 software. A combined glass electrode was employed for potential measurements. A standardized 0.1 M NaOH solution was used as the titrant. Each measurement day, the electrode was calibrated by triplicate titrations of a 0.1 M HClO_4_ solution of constant ionic strength (0.1 M NaClO_4_) using the standardized NaOH solution. Electrode parameters, namely *E*° and the slope factor, were determined using the GLEE software.^43^ All measurements were performed at a constant temperature of 25 °C under an inert argon atmosphere. Solutions were stirred magnetically throughout the experiments.

At least three titrations were performed for each ligand (*C*_L_ = 1 mM, pH range of 2–11). The purity and exact concentration of the ligand solution were determined using the Gran method.^44^ Potentiometric data were processed using the SUPERQUAD program, which employs nonlinear least-squares fitting methods.^45^ Stepwise protonation constants of the ligand were derived from the overall stability constants determined by the program and defined by eqs ([Disp-formula eq1]) and ([Disp-formula eq2]). Since complex formation occurs at pH < 2, which is outside the reliable working range of potentiometry and as this method requires notably higher analyte concentrations than UV–vis spectroscopy, stability constants of the complexes were determined spectrophotometrically.

## Results and Discussion

### Acid–Base and Spectroscopic Properties of Ligands

To characterize the acid–base properties of the ligands, a combination of potentiometry and UV–vis spectroscopy was employed, enabling comprehensive characterization over the entire pH range in which the functional groups of the ligands undergo deprotonation. The acid dissociation constants (p*K*_a_) for all ligands are collected in Table 1, and the proposed deprotonation sequence is shown in Figure 2 for **Ang**, taken as a representative example that contains the full set of protonation sites.

**Figure 2:**
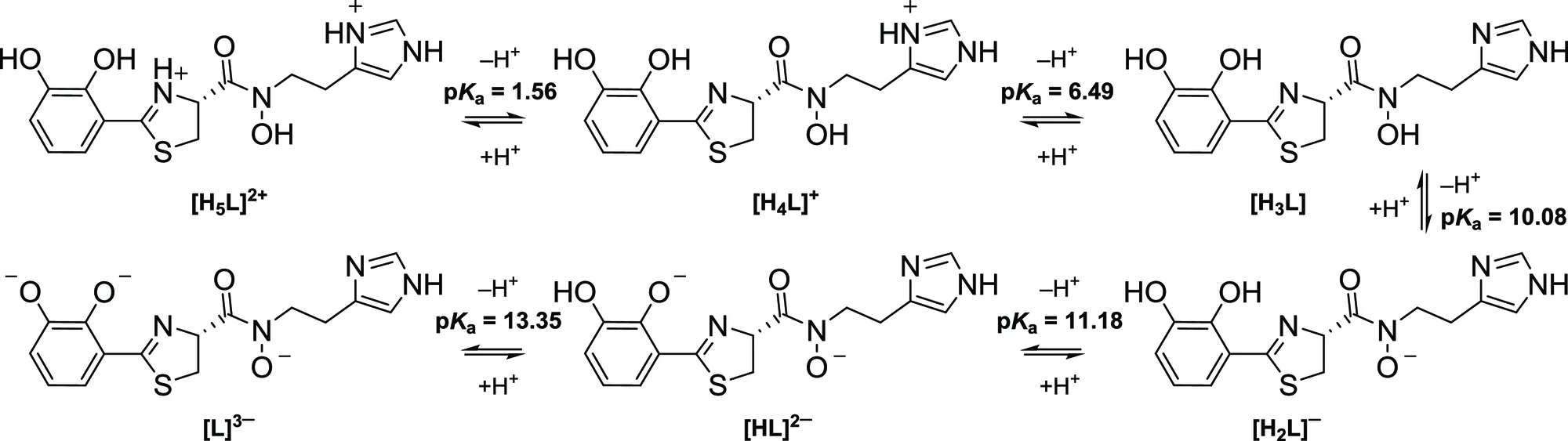
Stepwise deprotonation of the metal-free **Ang**.

**Table 1:** Overall Stability Constants (β) and Acid Dissociation Constants (p*K*_a_) of **L1**–**L4** and **Ang** in Solution^a^

	L1		L2		L3		L4		Ang	
	log β^b^	p*K*_a_ ^c^	log* *β^b^	p*K*_a_ ^c^	log* *β^b^	p*K*_a_ ^c^	log β^b^	p*K*_a_ ^c^	log β^b^	p*K*_a_ ^c^
[H_5_L]									42.66	1.56(21)^d^
[H_4_L]					30.22	1.33(36)^d^	35.20(11)^d^	1.13	41.10	6.49(4)^e^
[H_3_L]	19.40	1.65(14)^d^	22.47(23)^d^	1.36	28.89	4.72(6)^e^	34.07(9)^d^	9.68	34.61	10.08(2)^e^
[H_2_L]	17.75(2)^d^	4.94	21.11(23)^d^	8.92	24.17(18)^d^	10.82	24.39(5)^d^	11.04	24.53(1)^d^	11.18
[HL]	12.81(7)^d^	12.81	12.19(8)^d^	12.19	13.35(7)^d^	13.35	13.35(14)^d^	13.35	13.35	13.35

a Solvent: MeOH/H_2_O mixture (80:20 w/w), *I* = 0.1 M NaClO_4_, *T* = 25 °C. The reported errors are given as σ. Charges are omitted for clarity.

b Overall stability constants (β) expressed by the equation: β­(H_*n*_L) = [H_*n*_L]/([H^+^]^*n*^[L]).

c Acid dissociation constants (p*K*_a_) expressed by p*K*_a_ = log β­(H_*n*_L) – log β­(H_*n*–1_L).

d Calculated from pH-dependent UV–vis titrations.

e Calculated from potentiometric titrations.

The thiazoline ring nitrogen atom exhibits the lowest p*K*_a_ value of 1.13–1.65 (Table 1). The deprotonation of the carboxylic group occurs in the p*K*_a_ range of 4.72–4.94. An additional p*K*_a_ for **Ang** was attributed to the deprotonation of the imidazole ring nitrogen atom (p*K*_a_ = 6.49). The deprotonation of the hydroxamic groups appears in the p*K*_a_ range of 8.92–10.08. The acid dissociation constants of **L1** and **L2**, 12.81 and 12.19, respectively, were attributed to the deprotonation of the hydroxyphenyl group. For ligands **L3**, **L4**, and **Ang** containing a catechol moiety the p*K*_a_ values of 10.82–11.18 and p*K*_a_ of 13.35 were assigned to the deprotonation of the catechol hydroxyl groups. Due to the excellent agreement of p*K*_a_ of 13.35 for **L3** and **L4**, the determined deprotonation constant was treated as a fixed value for **Ang**. Taking into account various solvent and ionic strength conditions, the determined values of deprotonation constants are consistent with previous data for each type of functional group, i.e. the thiazoline ring,^28,46^ the carboxylic group,^13,28^ the imidazole ring,^47–49^ the hydroxamic group,^49–52^ the hydroxyphenyl group,^13,28,29^ and the catechol hydroxyl groups.^1^ Moreover, the deprotonation in deferitazole, an analog structurally closely related to **L1** and **L3**, proceeds successively at the thiazoline ring (p*K*_a_ of 1.7), the carboxylic group (p*K*_a_ of 3.8), and then at the hydroxyphenyl group (p*K*_a_ of 10.7).^53^ The deprotonation of the hydroxyphenyl group occurs with a higher p*K*_a_ than previously reported for **L1** (H_2_dad, p*K*_a_ of 10.47), which is attributable to different measurement conditions.^13^

For **L1** the electronic absorption spectrum of [H_3_L]^+^ is characterized by two main bands centered at 350 nm (ε_350_ = 6.0 × 10^3^ M^–1^ cm^–1^) and 275 nm (ε_275_ = 15.2 × 10^3^ M^–1^ cm^–1^), respectively (Figure S5a and Table S1). These two absorption bands most likely originate from the lowest allowed π–π* transitions^46,54,55^ which are derived from ^1^B_2u_→^1^A_1g_ (^1^L_b_ in Platt’s notation) and ^1^B_1u_ →^1^A_1g_ (^1^L_a_ in Platt’s notation) transitions of benzene, respectively.^56,57^ Both absorption bands are substantially bathochromically shifted with respect to related phenolate-based systems,^58–61^ reflecting the electronic effect of the ortho-thiazoline substituent.^28,60^ After an increase in pH and the formation of [H_2_L], these two absorption bands undergo strong hypsochromic and hypochromic shifts; the band with λ_max_ = 315 nm is characterized by ε_315_ = 4.6 × 10^3^ M^–1^ cm^–1^, and the band with λ_max_ ∼ 252 nm shows a split maximum (ε_254_ = 11.2 × 10^3^ M^–1^ cm^–1^; ε_251_ = 11.3 × 10^3^ M^–1^ cm^–1^). These spectral variations could be ascribed to the deprotonation of the imine-type nitrogen of the thiazoline ring.^28^ Under more basic conditions, deprotonation of [H_2_L] species to [HL]^−^, results in very weak spectral changes, demonstrating that the phenol-thiazoline chromophore is unaffected and that this deprotonation equilibrium is associated with the carboxylic acid functionality. The UV–vis spectrum of the [L]^2–^ species is characterized by bathochromic shifts of both bands. The band with λ_max_ = 258 nm displays a hypochromic effect (ε_258_ = 7.7 × 10^3^ M^–1^ cm^–1^), whereas the former undergoes a significant increase in molar absorptivity (ε_348_ = 8.8 × 10^3^ M^–1^ cm^–1^), changes consistent with deprotonation at the phenolate group.^58^ The spectroscopic characteristics of **L2** (Figure S5b and Table S1) is similar to those of **L1**. Deprotonation of the hydroxamic group (from [H_2_L] to [HL]^−^) also results in very weak spectral changes and does not affect the phenol-thiazoline chromophore, while deprotonation of phenolic moiety (from [HL]^−^ to [L]^2–^) occurs with a bathochromic (∼50 nm) and hyperchromic shift (ε_355_ ∼ 1.7 × 10^3^ M^–1^ cm^–1^).

For **L3**, which is the catechol analog of **L1**, the electronic absorption spectrum of [H_4_L]^+^ is characterized by a main band centered at 297 nm (ε_297_ = 10.1 × 10^3^ M^–1^ cm^–1^) (Figure S5c and Table S1), which can be assigned to the S_0_→S_2_ (π–π*) lowest lying energy transition of catechol.^62^ Similarly to the previously described ligands, the absorption band is bathochromically shifted relative to related catecholate systems, reflecting the electronic influence of the ortho-thiazoline substituent (with respect to C2). After an increase in the pH and the [H_3_L] formation, the band undergoes hypsochromic and hypochromic shift (λ_max_ = 265 nm, ε_265_ = 7.7 × 10^3^ M^–1^ cm^–1^), which could be ascribed to the deprotonation of the imine-type nitrogen of the thiazoline ring.^28^ Under more basic conditions, deprotonation of [H_3_L] species to afford [H_2_L]^−^, results in an increase in absorption intensity (Figure S5c), demonstrating that the catechol-thiazoline chromophore is unaffected and that this deprotonation equilibrium is associated with the carboxylic acid functionality. The UV–vis spectrum of the [HL]^2–^ species is characterized by two absorption bands with λ_max_ = 276 nm (ε_276_ = 11.3 × 10^3^ M^–1^ cm^–1^) and λ_max_ = 240 nm (ε_240_ = 20.5 × 10^3^ M^–1^ cm^–1^). These spectral variations could be ascribed to the deprotonation of the hydroxyl group of catecholate unit. Finally, upon deprotonation of [HL]^2–^ to [L]^3–^, a broad band with λ_max_ = 302 nm (ε_302_ = 20.5 × 10^3^ M^–1^ cm^–1^) arises from the overlap of two electronic transitions, as a consequence of deprotonation of the second hydroxyl group of the catecholate moiety. No polymeric species were observed, such species typically exhibit a band at 320 nm in aerated aqueous solution, which further confirms that appropriate anaerobic conditions were maintained throughout the measurements.^63^ **L4** is the catechol analog of **L2** and the hydroxamic analog of **L3**. The deprotonation of hydroxamic group ([H_3_L]→[H_2_L]^−^) in **L4** results in very weak spectral changes (Figure S5d and Table S1) and does not affect the catechol-thiazoline chromophore, while the deprotonation of the second hydroxyl group in catecholate moiety (from [HL]^2–^ to [L]^3–^) results in a broad composite band originating from the superposition of multiple electronic transitions.

In the spectrum of **Ang** (Figure S5e), the fully protonated species [H_5_L]^2+^ is characterized by high intensity band with λ_max_ = 294 nm (ε_294_ = 21.4 × 10^3^ M^–1^ cm^–1^, Table S1). Deprotonation of the imine-type nitrogen of the thiazoline ring (from [H_5_L]^2+^ to [H_4_L]^+^)^28^ results in a hypsochromic and hypochromic shift (λ_max_ = 264 nm, ε_264_ = 17.9 × 10^3^ M^–1^ cm^–1^), while subsequent deprotonation of the nitrogen of imidazole ring (from [H_4_L]^+^ to [H_3_L]) led only to a slight hyperchromic shift (λ_max_ = 263 nm, ε_263_ = 18.9 × 10^3^ M^–1^ cm^–1^). The formation of [H_2_L]^−^ species, which is a consequence of deprotonation of the hydroxamic group, results in a minor hypsochromic (∼3 nm) and a hyperchromic (∼3.2 × 10^3^ M^–1^ cm^–1^) shift. The last electronic spectrum of **Ang**, which consists of two bands with λ_max_ = 272 nm (ε_272_ = 19.7 × 10^3^ M^–1^ cm^–1^) and λ_max_ = 244 nm (ε_244_ = 32.9 × 10^3^ M^–1^ cm^–1^) corresponds to the ligand with one deprotonated hydroxyl group of the catecholate moiety ([HL]^2–^) and is analogous to the spectra of [HL]^2–^ species of ligands **L3** and **L4** (Figure S5c,d).

### Stoichiometry of Complexes with Fe­(III)

ESI-MS was used to determine the stoichiometry of Fe­(III) complexes formed with anguibactin and its analogs. The formation of 1:2 metal-to-ligand complexes was determined for all ligands (Figures S6–S11). For **Ang**, both 1:1 and 1:2 metal complexes have been observed. The data are presented in Table 2, and the mass spectra are provided in the Supporting Information. The spectrum recorded for a solution of Fe­(III)–**Ang** featured two peaks corresponding to mono- and bis-ligand complexes: {[HL]^2–^+Fe^3+^}^+^ (*m*/*z* = 402.0030) and {2­[H_2_L]^−^+Fe^3+^}^+^ (*m*/*z* = 750.0909) (Table 2 and Figure S11). The free ligand exhibited a peak at *m*/*z* = 349.0923, assigned to the [H_4_L]^+^ species. The occurence of 1:1 ^16^ and 1:2 ^19^ metal-to-ligand stoichiometry is consistent with the previously reported results for Fe­(III)–**Ang** complexes and depends on pH, with increasing pH favoring the formation of {2­[H_2_L]^−^+Fe^3+^}^+^ species over {[HL]^2–^+Fe^3+^}^+^. Additionally, a low-intensity peak (∼1.2 × 10^6^) corresponding to {2­[HL]^2–^+[H_2_L]^−^+2Fe^3+^}^+^ (*m*/*z* = 1151.0864) form was observed. The dominant form is 1:2 (an intensity of ∼4.6 × 10^6^), which is analogous to that observed for other ligands **L1–L4** (Table 2, and Figures S6–S11). The protonation state observed by ESI-MS may differ from that present in solution due to proton transfer processes occurring during ionization and in the gas phase.

**Table 2:** Experimental^a^ and Calculated *m*/*z* Values Observed for Fe­(III) Complexes with **L1–L4** and **Ang**

Ligand	*m*/*z* experimental	*m*/*z* calculated	Assignment	Molecular formula
L1	224.0358	224.0376	[H_3_L]^+^	C_10_H_10_NO_3_S^+^
	499.9759	499.9794	{2[HL]^−^+Fe^3+^}^+^	C_20_H_16_FeN_2_O_6_S_2_^+^
L2	239.0482	239.0485	[H_3_L]^+^	C_10_H_11_N_2_O_3_S^+^
	529.9975	530.0012	{2[HL]^−^+Fe^3+^}^+^	C_20_H_18_FeN_4_O_6_S_2_^+^
L3	240.0306	240.0325	[H_4_L]^+^	C_10_H_10_NO_4_S^+^
	531.9655	531.9692	{2[H_2_L]^−^+Fe^3+^}^+^	C_20_H_16_FeN_2_O_8_S_2_^+^
L4	255.0417	255.0434	[H_4_L]^+^	C_10_H_11_N_2_O_4_S^+^
	561.9870	561.9910	{2[H_2_L]^−^+Fe^3+^}^+^	C_20_H_18_FeN_4_O_8_S_2_^+^
Ang	349.0923	349.0965	[H_4_L]^+^	C_15_H_17_N_4_O_4_S^+^
	402.0030	402.0080	{[HL]^2–^+Fe^3+^}^+^	C_15_H_14_FeN_4_O_4_S^+^
	750.0909	750.0972	{2[H_2_L]^−^+Fe^3+^}^+^	C_30_H_30_FeN_8_O_8_S_2_^+^
	1151.0864	1151.0981	{2[HL]^2–^+[H_2_L]^−^+2Fe^3+^}^+^	C_45_H_43_Fe_2_N_12_O_12_S_3_^+^

a *C*_Fe(III)_ = 0.1 mM, Solvent: MeOH/H_2_O mixture (80:20 w/w), M/L = 1:2 for **L1**, **L2** and **Ang**, and 1:3 for **L3** and **L4**.

### Binding Characteristics of Fe­(III) Complexes

The interactions of **Ang** and its analogs with Fe­(III) were investigated by pH-dependent spectrophotometric titrations (Figure 3). Analogs **L1** and **L2** form [FeL]^+^ complexes that predominate up to pH ∼ 3 (Figure S12a,b). These species exhibit ligand-to-metal charge transfer (LMCT) bands with λ_max_ = 540 nm (ε_540_ = 1.6 × 10^3^ M^–1^ cm^–1^, Figure 3a,b, and Table 3) and λ_max_ = 417 nm (ε_417_ = 1.7 × 10^3^ M^–1^ cm^–1^) which, per analogy with Fe­(III)–pyochelin complexes, can be attributed to phenolate→Fe­(III) charge transfers, assigned to a transition from π_1_ (HOMO orbital of π symmetry) to an iron d_π_ (*t*_2g_) orbitals.^28,64^ In **L1**, [FeL]^+^ species most likely corresponds to the coordination of the Fe­(III) ion by three donor atoms: the nitrogen atom of the thiazoline ring (N_thz_), oxygen atom of the hydroxyphenyl moiety (O_ph_), and oxygen atom of the carboxylate (O_cbx_):{N_thz_, O_ph_, O_cbx_}, similarly to AA in complexes with Fe­(III) and Al­(III) ions.^14,65^ In **L2** the carboxylate donor is replaced by the hydroxamate group (O_hx_):{N_thz_, O_ph_, O_hx_}. The corresponding stability constants (log β) are 22.84(17) for Fe­(III)–**L1** and 24.93(14) for Fe­(III)–**L2**, respectively (Table 4). At higher pH, the [FeL_2_]^−^ species becomes dominant. Binding of the second ligand results in a hypsochromic and hyperchromic shifts (λ_max_ = 485 nm; ε_485_ = 4.6 × 10^3^ M^–1^ cm^–1^, and a shoulder at ∼412 nm with ε_412_ = 5.2 × 10^3^ M^–1^ cm^–1^ for **L1** and λ_max_ = 492 nm; ε_492_ = 4.3 × 10^3^ M^–1^ cm^–1^, λ_max_ = 412 nm; ε_412_ = 4.0 × 10^3^ M^–1^ cm^–1^ for **L2**) and allows complete saturation of the Fe­(III) coordination sphere, 2 × {N_thz_, O_ph_, O_cbx_} and 2 × {N_thz_, O_ph_, O_hx_} and the formation of stable complexes with log β of 39.59(21) and 44.11(21), respectively. The proposed coordination modes have been observed in the crystal structures of of FemA loaded with Fe­(III)–AA_2_ and Fe­(III)–DHA_2_, with the key interactions being conserved between FemA and these two complexes.^14^ These results show that the 1:1 Fe­(III)–DHA and Fe­(III)–AA ratio proposed earlier, with coordination through the phenolate oxygen alone, seems incorrect and our data extend those studies with a complete coordination model, enabling a rigorous assessment of the complexes’ thermodynamic stability.^15^

**Figure 3:**
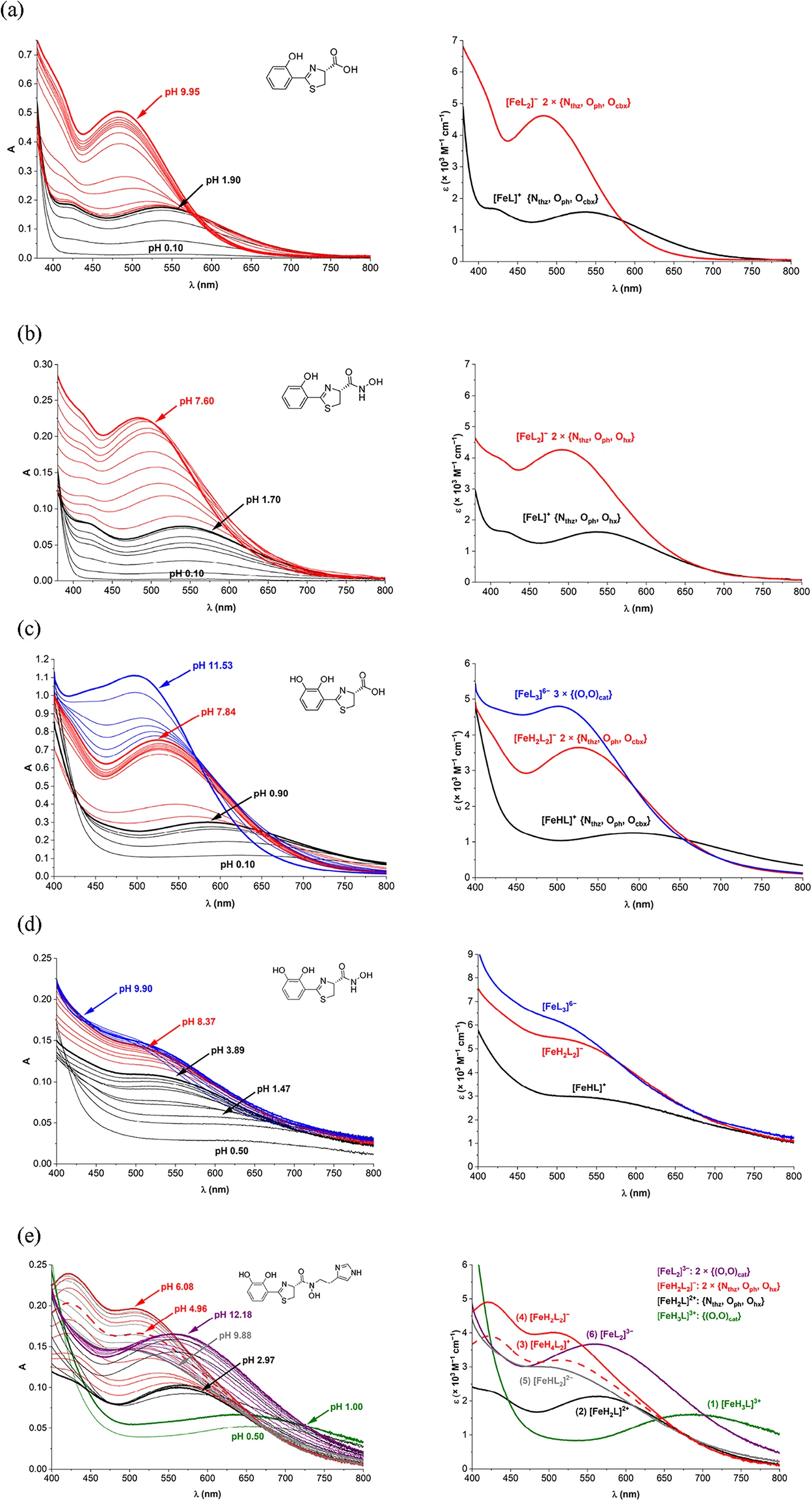
Absorption and electronic spectra of Fe­(III) complexes with (a) **L1**, (b) **L2**, (c) **L3**, (d) **L4**, and (e) **Ang**. *C*_L_ range of 0.5–5 × 10^–4^ M; for **L1**, **L2** and **Ang** M/L = 1:2, for **L3** and **L4** M/L = 1:4. Solvent: MeOH/H_2_O mixture (80:20 w/w), *I* = 0.1 M NaClO_4_, *T* = 25 °C.

**Table 3:** Electronic Absorption Spectral Data for Fe­(III) Complexes with **L1**–**L4** and **Ang** in Solution^a^

	L1		L2		L3		L4		Ang	
	λ_max_ [nm]	ε [×10^3^ M^–1^ cm^–1^]	λ_max_ [nm]	ε [×10^3^ M^–1^ cm^–1^]	λ_max_ [nm]	ε [×10^3^ M^–1^ cm^–1^]	λ_max_ [nm]	ε [×10^3^ M^–1^ cm^–1^]	λ_max_ [nm]	ε [×10^3^ M^–1^ cm^–1^]
[FeH_3_L]									690	1.6
[FeH_2_L]									563	2.1
									420	2.3
[FeHL]					590	1.2	530	3.0		
[FeL]	540	1.6	540	1.6						
	417	1.7	417	1.6						
[FeH_4_L_2_]									518	3.2
									420	3.9
[FeH_2_L_2_]					530	3.5	510	5.4	510	4.0
									420	4.9
[FeHL_2_]									503	2.4
[FeL_2_]	485	4.6	492	4.3					560	3.7
	412^sh^	5.2	412	4.0						
[FeL_3_]					500	5.3	500	6.2		

a Solvent: MeOH/H_2_O mixture (80:20 w/w), *I* = 0.1 M NaClO_4_, *T* = 25 °C. Experimental errors: λ_max_ = ±2 nm, ε = ±5%. Charges are omitted for clarity.

**Table 4:** Stability Constants (log β) of Fe­(III) Complexes with **L1**–**L4** and **Ang** in Solution^a^

	L1	L2	L3	L4	Ang
	log β^b^	log β^b^	log β^b^	log β^b^	log* *β^b^
[FeH_3_L]					46.17(4)
[FeH_2_L]					45.01(4)
[FeHL]			33.78(16)	38.04(10)	
[FeL]	22.84(17)	24.93(14)			
[FeH_4_L_2_]					82.28(7)
[FeH_2_L_2_]			61.93(18)	69.22(16)	72.06(12)
[FeHL_2_]					63.89(12)
[FeL_2_]	39.59(21)	44.11(21)			52.73(12)
[FeL_3_]			53.23(28)	66.66(53)	

a Solvent: MeOH/H_2_O mixture (80:20 w/w), *I* = 0.1 M NaClO_4_, *T* = 25 °C.

b Overall stability constants (β) expressed by the equation: β­(M_*p*_H_*q*_L_*r*_) = [M_*p*_H_*q*_L_*r*_]/([M]^*p*^[H^+^]^*q*^[L]^*r*^). The reported errors are given as σ. Charges are omitted for clarity.

Since ligands **L3**, **L4**, and **Ang** contain a catechol moiety capable of saturating the Fe­(III) coordination sphere with three ligand molecules, a 1:4 metal-to-ligand molar ratio was employed in the spectroscopic studies of **L3** and **L4** in order to fully characterize such complexes and to exclude their formation for Fe­(III)–**Ang**. In the spectra of Fe­(III)–**L3** complex, first LMCT band with λ_max_ = 590 nm is observed (Figure 3c), corresponding to the [FeHL]^+^ species, with coordination most likely analogous to that in [FeL]^+^ species of **L1** ligand, i.e., via hydroxyphenyl-thiazoline and carboxylate moiety, {N_thz_, O_ph_, O_cbx_}. The bathochromic shift observed for the catechol analog relative to the phenol analogs is consistent with the electron-donating effect of the additional hydroxyl group, which raises the energy of the ligand π donor orbitals and lowers the LMCT transition energy, even when this group is not directly involved in Fe­(III) coordination.^66^ The absorption band of the main species at pH 7 (Figures 3c and S12c), [FeH_2_L_2_]^−^, shows λ_max_ = 530 nm (ε_530_ = 3.5 × 10^3^ M^–1^ cm^–1^). This species forms when a second ligand molecule binds to the metal ion, most likely through the same donor set, 2 × {N_thz_, O_ph_, O_cbx_}, thereby completing the coordination sphere of the Fe­(III) ion with six donor atoms. Under basic conditions **L3** is additionally capable of coordinating Fe­(III) through the catechol oxygen donors of three ligand molecules, giving a tris­(catecholate)-type, 3 × {(O,O)_cat_}, coordination sphere in [FeL_3_]^6–^ species, as confirmed by the presence of a band at λ_max_ = 500 nm (ε_500_ = 5.3 × 10^3^ M^–1^ cm^–1^).^67–71^ Corresponding stability constants are given in Table 4. **L4** was expected to provide the closest synthetic reference for the Fe­(III)–**Ang** system. Its spectral behavior, however, somehow differs from that of the remaining ligands. In the Fe­(III)–**L4** complex (Figure 3d), the spectra are broad and considerably less resolved than the corresponding bands of Fe­(III)–**L3**. The complexes were also the least soluble of the series, and the spectra display a pronounced baseline offset. A preliminary model accounting for the pH-dependent spectral changes requires three species, with calculated molar absorption spectra with maxima at 530, 510, and 500 nm. However, the donor sets underlying these species cannot be assigned unambiguously from the absorption data alone and may include mixed catecholate–hydroxamate coordination.^67^ Unlike for the Fe­(III)–**L3** complex, the band at 500 nm (assigned to a tris-catecholate mode) starts to decrease already around pH 9.9, consistent with possible dissociation of the complex. In view of this divergent behavior, the Fe­(III)–**L4** speciation model is regarded as preliminary and treated with caution.

**Ang** forms the most complex pH-dependent system with Fe­(III) ions. At very low pH, a band at λ_max_ = 690 nm (ε_690_ = 1.6 × 10^3^ M^–1^ cm^–1^, Figure 3e) and a green color of the Fe­(III)–**Ang** solution were observed. These correspond to the first species, [FeH_3_L]^3+^ (Figure 4), in which Fe­(III) ion is chelated by a single catecholate group.^67–70^ This form must nevertheless be treated with considerable caution, as its abundance is negligible and it is observed only under very acidic conditions.

**Figure 4:**
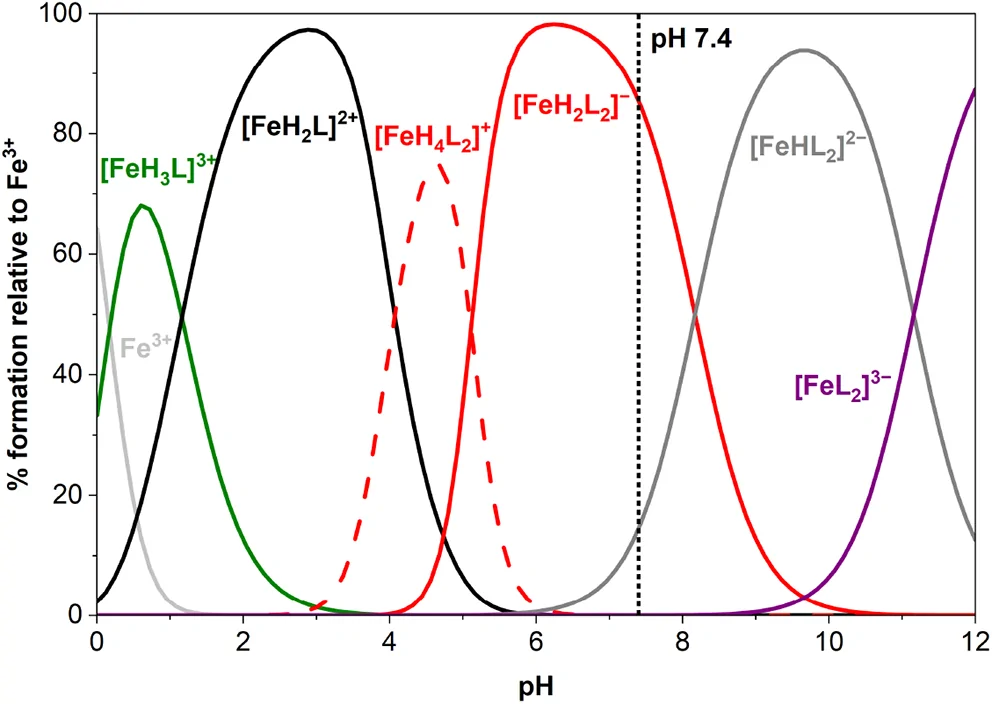
Distribution plot of the Fe­(III)–**Ang** complexes as a function of pH. The color coding of the species corresponds to that used in Figure 3e; solvent: MeOH/H_2_O mixture (80:20 w/w), *i* = 0.1 M NaClO_4_, *T* = 25 °C. [L]_tot_ = 2 × 10^–4^ M; [Fe­(III)]_tot_/[L]_tot_ = 0.5.

As pH increases, new LMCT bands arise, with λ_max_ at ∼563 and 420 nm (ε_563_ = 2.1 × 10^3^ M^–1^ cm^–1^, ε_420_ = 2.3 × 10^3^ M^–1^ cm^–1^, Figure 3e, and Table 3). The thiazoline ring deprotonates (p*K*_a_ = 1.16) and in [FeH_2_L]^2+^ the coordination changes to a hydroxyphenyl-thiazoline mode, {N_thz_, O_ph_, O_hx_}, (log β of 45.01(4), Table 4; schematic structure in Figure 5), analogous to the previously described complexes of the **L1** and **L2**. Above pH ∼ 3, the LMCT band is blue-shifted to 518 nm (ε_518_ = 3.2 × 10^3^ M^–1^ cm^–1^), and the change is assigned to the formation of [FeH_4_L_2_]^+^ species, with M/L stoichiometry of 1:2, 2 × {N_thz_, O_ph_, O_hx_} binding mode with two imidazole rings in protonated form (and therefore most likely noncoordinating, Figure 5). The band at 420 nm is increasing in intensity (ε_420_ = 3.9 × 10^3^ M^–1^ cm^–1^). Further increase of intensity of both bands up to pH ∼ 6 (ε_510_ = 4.0 × 10^3^ M^–1^ cm^–1^ and ε_420_ = 4.9 × 10^3^ M^–1^ cm^–1^) ascribed to the formation of [FeH_2_L_2_]^−^ species, arising from the deprotonation of both imidazoles’ ring nitrogens (p*K*_a_ ∼ 5.1). The above spectral characteristics is in very good agreement with the previous data for Fe­(III)-**Ang**, collected in aqueous solution at pH 7.5 (λ_max_ at 500 and 410 nm);^16^ [FeH_2_L_2_]^−^ predominates over pH range of 5.2–8.2 (Figure 4) and is characterized by log β = 72.06(12). Notably, Fe­(III) strongly prefers phenol-oxazoline-hydroxamate chelation, and the imidazole nitrogen is therefore unlikely to coordinate directly, it may nevertheless affect the stability of the complexes through indirect interactions, as observed for related imidazole-containing hydroxamic acids.^49^ Above pH = 6.1, another change in the spectral behavior is observed: a decrease of the intensity of LMCT band at 518 nm with a disappearance of LMCT band at 420 nm. This, above pH 9.88 is further followed by the formation of another band–with λ_max_ at 560 nm (Figure 3e). The changes correspond to the modifications of the coordination sphere, in which the previously mentioned binding groups are progressively replaced by one catecholate group ([FeHL_2_]^2–^) and two catecholate groups ([FeL_2_]^3–^). In [FeHL_2_]^2–^ we propose one **Ang** being coordinated to Fe­(III) via {N_thz_, O_ph_, O_hx_, N_im_}, and the second via {(O,O)_cat_} (Figure 5). Coordination through the {N_thz_, O_ph_, O_hx_, N_im_} donors was already observed in the Ga­(III) complex of **Ang** in the solid state – the use of excess Ga­(III) afforded a dinuclear complex (M/L = 2:2), with solvent molecules completing the coordination sphere of each metal center.^18^ Asymmetric coordination through different donor atoms of two identical ligand molecules has also been described for the Fe­(III) and Ga­(III) complexes of pyochelin, in which one ligand contributes four donor atoms: {N_thz_, O_ph_, N_thiazolidine_, O_cbx_} and the second completes the coordination sphere with two further donors: {N_thz_, O_ph_}.^28,72^ Moreover, a mixed coordination motif with the same set of donor atoms as proposed here for Fe­(III)–**Ang** was found in the crystal structure of the ferric preacinetobactin–acinetobactin complex bound to the BauA transporter (PDB 6H7F, Figure 6).^3,73^ The final [FeL_2_]^3–^ species exhibits a single broad band at 560 nm (ε_560_ = 3.7 × 10^3^ M^–1^ cm^–1^, a violet color of solution), diagnostic of a bis-catecholate Fe­(III) coordination environment (Figure 5).^69–71,74^ Coordination of Fe­(III) by two catecholate moieties together with two additional imidazole rings is an uncommon binding motif, but it is not without precedent among natural siderophores: acinetobactin is able to form a bis-tridentate (M/L = 1:2) complex in which each ligand supplies one catecholate and one imidazole donor to the Fe­(III) ion, as established by the crystal structure of the Fe­(III)–acinetobactin complex bound to its periplasmic binding protein BauB (PDB 6MFL, Figure 6).^3,75^ The possibility of an analogous additional stabilization of the Fe­(III) ion at high pH by the imidazole moiety of **Ang** should therefore be considered. The formation of a mononuclear tris-catecholate (M/L = 1:3), as in [FeL_3_]^6–^ complexes of **L3** and **L4** ligands can be ruled out in this system.

**Figure 5:**
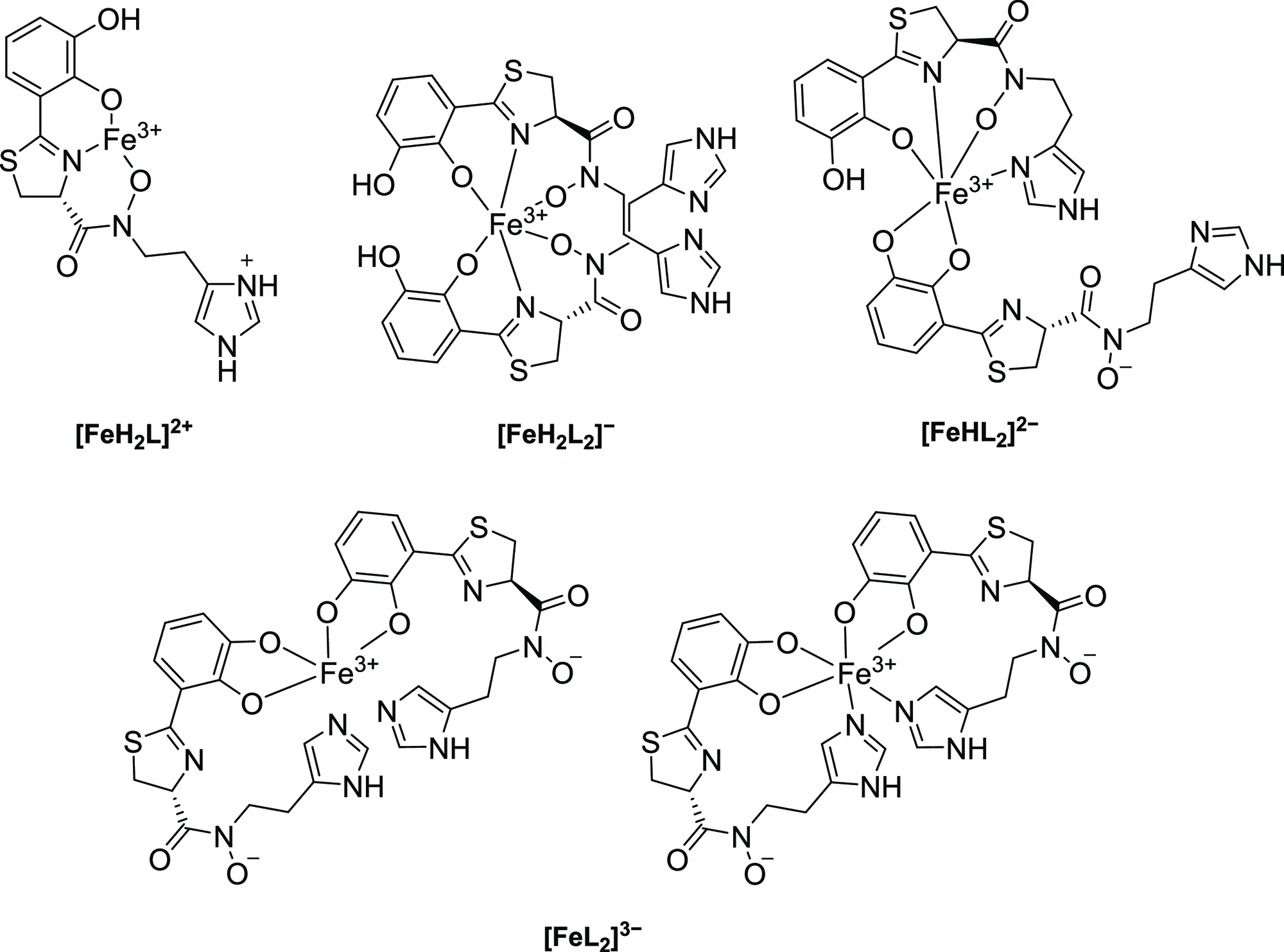
Proposed schematic structures of Fe­(III)–**Ang** complexes. Solvent molecules completing the octahedral coordination sphere are omitted for clarity.

**Figure 6:**
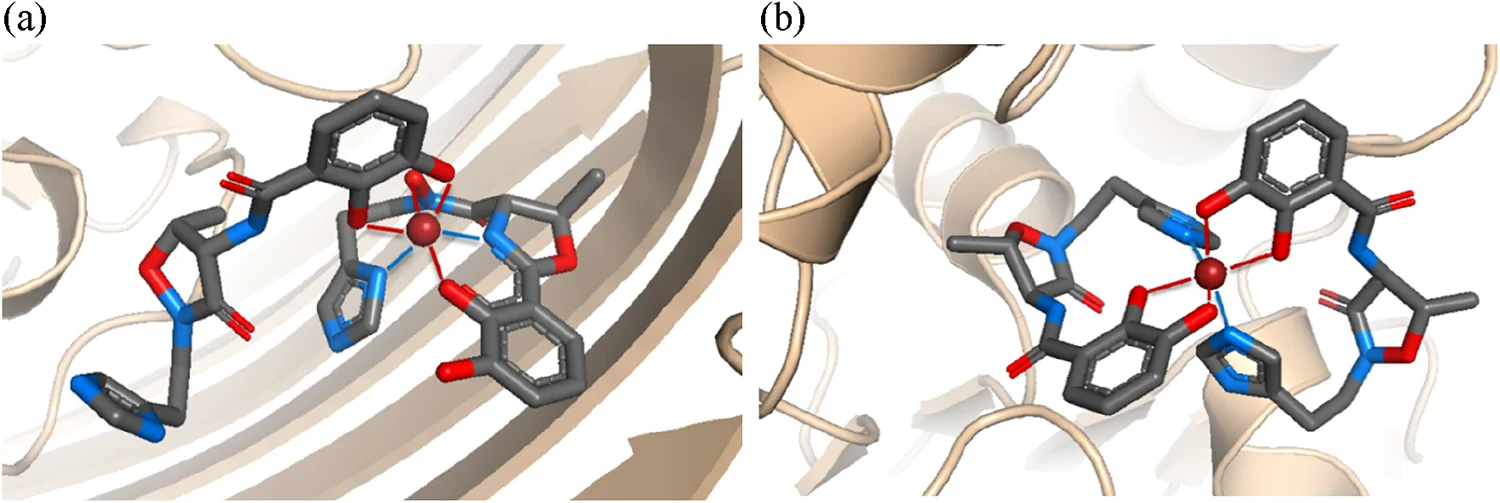
Structure of: (a) Fe­(preacinetobactin)­(acinetobactin) ternary complex (1:1:1) bound to protein BauA (PDB 6H7F);^73^ (b) Fe­(acinetobactin)_2_ complex bound to protein BauB (PDB 6MFL).^75^ Preacinetobactin and acinetobactin molecules are represented as sticks with carbon atoms colored dark gray, oxygen atoms in red and nitrogen atoms in blue. The Fe­(III) is represented as a firebrick sphere. Red and blue lines represent the coordination bonds. Reprinted from ref 3. Copyright (2026), with permission from Elsevier.

### Overall Stability and pM of Fe­(III) Complexes

The investigated ligands differ both in the aromatic ring substitution and in the auxiliary donor group attached to the thiazoline ring. Because these substitutions change the number and basicity of the protonation sites, the overall and stepwise stability constants are not directly comparable between ligands. The relative Fe­(III) affinities are therefore expressed as pFe values, where pFe = −log­[Fe­(III)]_free_, and *C*_L_ = 10 μM, *C*_Fe(III)_ = 1 μM at pH 7.4.^76^ All reported values refer to methanol/water (80:20 w/w) mixture used as a solvent and ionic strength *i* = 0.1 M NaClO_4_. The pFe values decrease in the order **Ang** (28.2) > **L2** (27.3) > **L4** (26.5) > **L1** (24.6) > **L3** (24.2), spanning 4 log units (Table 5). Two structural trends govern this ranking. First, replacing the carboxylate by the hydroxamate group increases the Fe­(III) affinity. pFe rises by 2.7 units from **L1** to **L2** (phenol series) and by 2.3 units from **L3** to **L4** (catechol series). This is consistent with the hydroxamate functioning as a stronger donor toward the Fe­(III) ion, identifying the auxiliary donor as the important determinant of affinity among the synthetic analogs.^77^ Second, and in contrast, exchanging the phenol for a catechol group does not enhance pFe. The carboxylate pair is essentially unchanged (**L1**, 24.6 and **L3**, 24.2), while within the hydroxamate pair the catechol analog lies 0.8 units below its phenol counterpart (**L4**, 26.5 vs **L2**, 27.3). The additional proton competition at pH 7.4 offsets the intrinsic stabilization otherwise expected from a fully chelating catecholate, accounting for the absence of any catechol-derived enhancement in pFe. **Ang** retains the highest Fe­(III) affinity of the series (pFe = 28.2), exceeding its closest synthetic analog **L4** by 1.7 units. Because **L4** reproduces the catechol and hydroxamate donor functionalities of the natural product but not its complete binding framework, this difference in stability and the higher pFe reflect the preorganization of the intact siderophore, possibly reinforced by additional stabilizing interactions such as π–stacking between the imidazole rings of the two coordinated ligands.^75,78^

**Table 5:** pM^a^ Values for Fe­(III) Complexes with **L1**–**L4** and **Ang** and Other Chelators

Ligand	pFe
**L1** ^b^	24.6
**L2** ^b^	27.3
**L3** ^b^	24.2
**L4** ^b^	26.5
**Ang** ^b^	28.2
DHA^c^	21.1 ^13,30^
Pyochelin^d^	16.0 ^28^
Desferrithiocin^e^	20.4 ^13^
	22.1 ^29^
Nordesferrithiocin^e^	20.5 ^29^
Deferitazole^e^	22.3 ^53^
L^d^	14.3 ^30^
L^d^	18.4 ^30^
L^d^	21.9 ^30^
Desferrioxamine B^f^	26.1 ^79^
Desferrioxamine E^c^	27.3 ^80^
Pyoverdine^f^	27.0 ^81^
Enterobactin^e^	35.5 ^82^

a pFe = −log­[Fe­(III)]_free_; *C*_L_ = 10 Μm; *C*_Fe(III)_ = 1 μM at pH 7.4.

b Solvent: MeOH/H_2_O mixture (80:20 w/w), *I* = 0.1 M NaClO_4_, *T* = 25 °C.

c Solvent: H_2_O, *I* = 0.1 M KNO_3_, *T* = 25 °C.

d Solvent: MeOH/H_2_O mixture (80:20 w/w), *I* = 0.1 M (C_2_H_5_)_4_NClO_4_, *T* = 25 °C.

e Solvent: H_2_O, *I* = 0.1 M KCl, *T* = 25 °C.

f Solvent: H_2_O, *I* = 0.1 M NaClO_4_, *T* = 25 °C.

Restricting the comparison to chelators characterized in the same solvent (CH_3_OH/H_2_O 80:20 w/w), **L1**–**L4** and **Ang** exceed the parent siderophore pyochelin (pFe = 16.0,^28^ Table 5), and the aromatic phenol-thiazole chelators of the BHPTC family: the monomeric L^1^ (14.3)^30^ and the bipodal L^2E^ (18.4)^30^ and L^2A^ (21.9).^30^ When comparing with ligands measured in aqueous solution, the pFe of **L1** differs by 3.5 units from the value reported previously for DHA (H_2_dad).^13,30^ It is also 2.3 units higher than the pFe value of the corresponding polyether derivative, deferitazole, containing the same phenol–thiazoline scaffold with an auxiliary carboxylate group.^53^ The comparison of **L1**, with AA is impossible as the only quantitative iron-binding information available is a dissociation constant obtained from fluorescence-quenching titrations in the methanol, which was derived assuming a 1:1 metal-to-ligand stoichiometry, and which the authors explicitly describe as an estimate rather than an absolute value.^15^ However, considering the information provided by structural studies which revealed that FemA is able to bind Fe­(III)–AA_2_ and Fe­(III)–DHA_2_ complexes in a very close manner and the key interactions are conserved between FemA and these two complexes,^14,65^ it is difficult to expect a significant difference in complex stability of the two ligands. Once placed in the broader context of well-characterized siderophores, the present scaffold clearly falls within high affinity regime. Of importance, a drastic increase of **Ang** affinity over that of pyochelin (ΔpFe > 12) can stem from different geometrical arrangements of the ligands in Fe­(III) complexes: in **Ang** being symmetrical with two ligands binding via the same motif, while in pyochelin asymmetrical–with one ligand playing a role of tetradentate chelator and the second completing the coordination sphere as a bidentate unit (*vide supra*). **Ang** affinity for Fe­(III) ions exceeds also that of desferrithiocin (pFe = 20.4 ^13^, 22.1 ^29^), which bears a phenolic hydroxyl group on the pyridine ring and a carboxylic acid group on the thiazoline ring, and is very close to those of the tris­(hydroxamate) siderophores desferrioxamine B (26.1 ^79^) and E (27.3 ^80^), as well as pyoverdine (27.0 ^81^), a peptide-based siderophore comprising dihydroxyquinoline, hydroxamate, and catecholate motifs. The affinity of **Ang** remains lower than the tris­(catecholate) reference enterobactin (35.5 ^82^).

## Conclusions

By studying **Ang** alongside a matched set of four synthetic analogs (**L1**–**L4**), in which the aromatic ring (phenol vs catechol) and the auxiliary donor (carboxylate vs hydroxamate) were varied independently, we were able to dissect the individual contributions of each chelating motif to the physicochemical properties of arylthiazoline chelators against an otherwise common structural background. To the best of our knowledge, this work provides only the second complete solution thermodynamic characterization of an arylthiazoline siderophore, after pyochelin, that is, one combining experimentally determined ligand protonation constants, a validated pH-dependent speciation model, and a pFe value calculated at pH 7.4 under the standard conditions. For **Ang**, neither the stability constant nor the pFe value have been reported to date.^3^ Across the series, ESI-MS and pH-dependent UV–vis titrations allowed to consistently identify bis-ligand complexes as the species that predominate at physiological pH. In these complexes the Fe­(III) ion is coordinated by two phenolate–thiazoline units acting together with the auxiliary carboxylate or hydroxamate donor, completing a six-coordinate, mixed N,O sphere. Coordination through the catecholate oxygens is not operative at pH 7.4 even for the catechol-bearing ligands. It becomes accessible only under basic conditions, where the catechol analogs **L3** and **L4** shift to a 1:3 (M/L) tris-catecholate mode. **Ang** behaves as the most elaborate member of this family, switching through a sequence of pH-dependent coordination modes and reaching its dominant [FeH_2_L_2_]^−^ form at physiological pH. The thermodynamic data reveal two clear structure–affinity relationships. The auxiliary donor is the principal determinant of Fe­(III) affinity among the synthetic analogs: replacing a carboxylate with a hydroxamate raises pFe in both the phenol (**L1** and **L2**) and catechol (**L3** and **L4**) pairs, consistent with the hydroxamate acting as the stronger donor group toward Fe­(III) ions. In contrast, substituting phenol for catechol provides no thermodynamic benefit at physiological pH, because the catechol engages iron through a single phenolate oxygen while its second hydroxyl only raises ligand basicity and increases proton competition. **Ang** retains the highest Fe­(III) affinity of the series (pFe = 28.2), arising from the presence of a strong donor set and, most likely, additional stabilizing interactions between the imidazole rings. The scaffold thus places **Ang** among high affinity siderophores, surpassing pyochelin and related phenol-thiazole chelators, close to tris­(hydroxamate) ferrioxamines, while remaining below the tris­(catecholate) benchmark enterobactin. These results provide the structure–property framework needed to interpret the coordination behavior of the arylthiazoline siderophore class and could be helpful for the future rational design of siderophore–antibiotic conjugates that exploit this scaffold. Since the imidazole nitrogen of **Ang** appears not to be directly engaged in Fe­(III) binding in the main species at physiological pH, it could serve as the point of derivatization and attachment of an antibiotic in siderophore–antibiotic conjugates targeting drug-resistant *A. baumannii*, with **Ang** acting as a more stable surrogate of acinetobactin.^19,24^ It should be noted, however, that the deprotonation constants of imidazoles and thus stabilization provided to the complex by their possible interaction, as well as solution speciation, may be markedly affected by structural modifications, and should be carefully designed.

## Supplementary Material



## Notes

NMR Spectra of **L2** and **L4**, UV–vis spectra of **L1**–**L4** and **Ang**, Mass spectra of Fe­(III) complexes with **L1**–**L4** and **Ang**, Distribution Plots of Fe­(III) complexes with **L1**–**L4** (PDF)
