# Supplementary material for: Arylthiazoline Unit as a Ferric Ion Chelating Moiety in Siderophores: The Coordination Chemistry of Anguibactin and Its Analogs
Source: Inorg Chem. 2026 Sep 1;65(36):21382–95. doi: 10.1021/acs.inorgchem.6c03994 (PMC13580194; doi:10.1021/acs.inorgchem.6c03994)
Supplement: Supplementary file 1 [file ic6c03994_si_001.pdf]

Supporting Information

Arylthiazoline unit as a ferric ion chelating moiety in siderophores: the coordination chemistry of anguibactin and its analogs

Klaudia Szczerba[a], Andrzej Mular[a], Karolina Piasta[a], Karolina Kamińska[b], Oscar Palacios[c], Mercè Capdevila[c], Elżbieta Wojaczyńska[b], and Elżbieta Gumienna-Kontecka[a,*]

[a] Faculty of Chemistry, University of Wrocław, F. Joliot-Curie 14, 50-383 Wrocław, Poland
[b] Faculty of Chemistry, Wrocław University of Science and Technology, Wybrzeże Wyspiańskiego 27, 50-370 Wrocław, Poland
[c] Departament de Química, Universitat Autònoma de Barcelona, 08193 Cerdanyola del Vallès, Spain
*Email: elzbieta.gumienna-kontecka@uwr.edu.pl

Table of Contents

1. NMR Spectra of L2 and L4 2

2. UV-Vis spectra of L1–L4 and Ang 6

3. Mass spectra of Fe(III) complexes with L1–L4 and Ang 8

4. Distribution Plots of Fe(III) complexes with L1–L4 14

1. NMR Spectra of L2 and L4

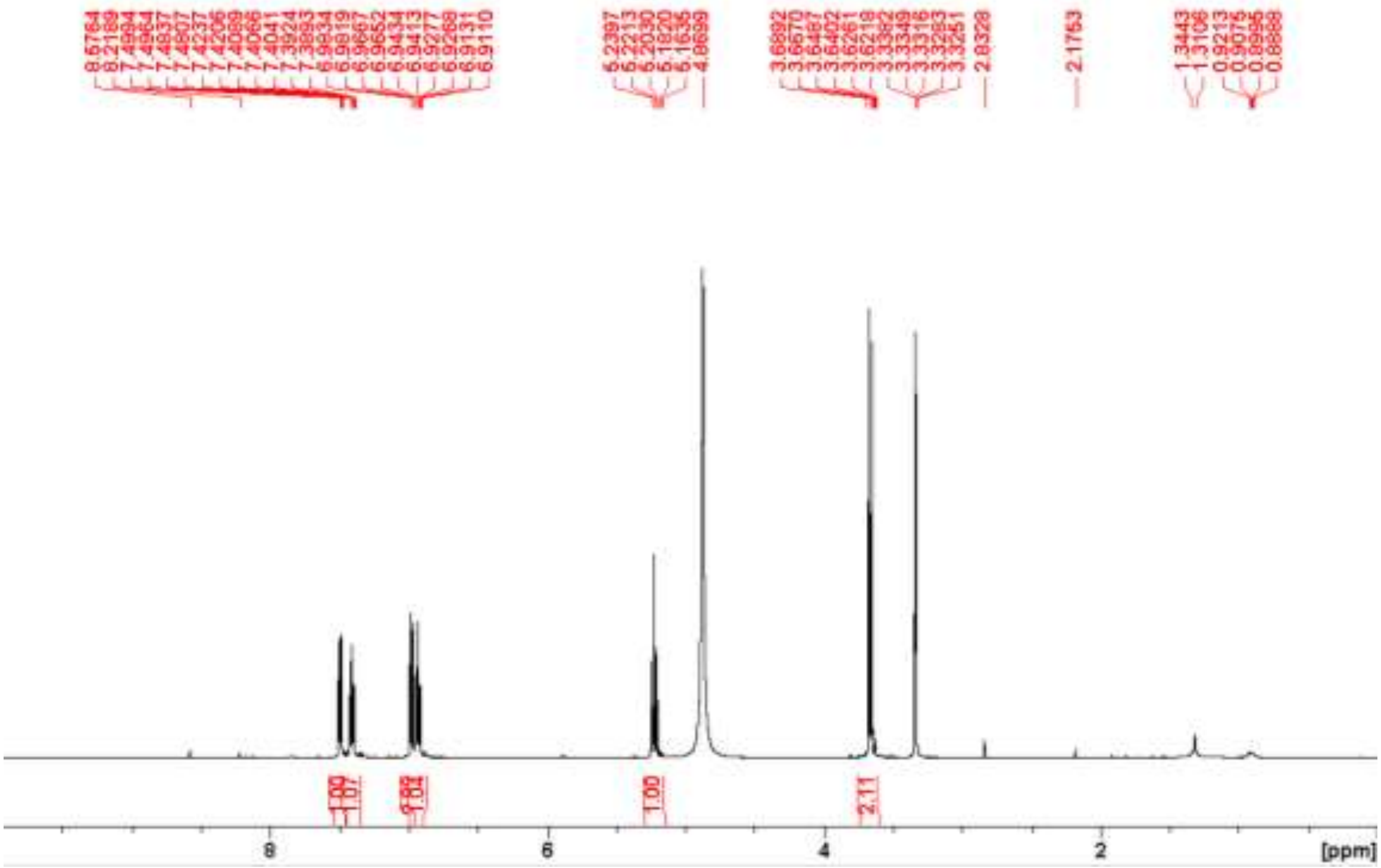

Figure S1. ^{1}H NMR spectrum of compound **L2** measured in CD_3OD.

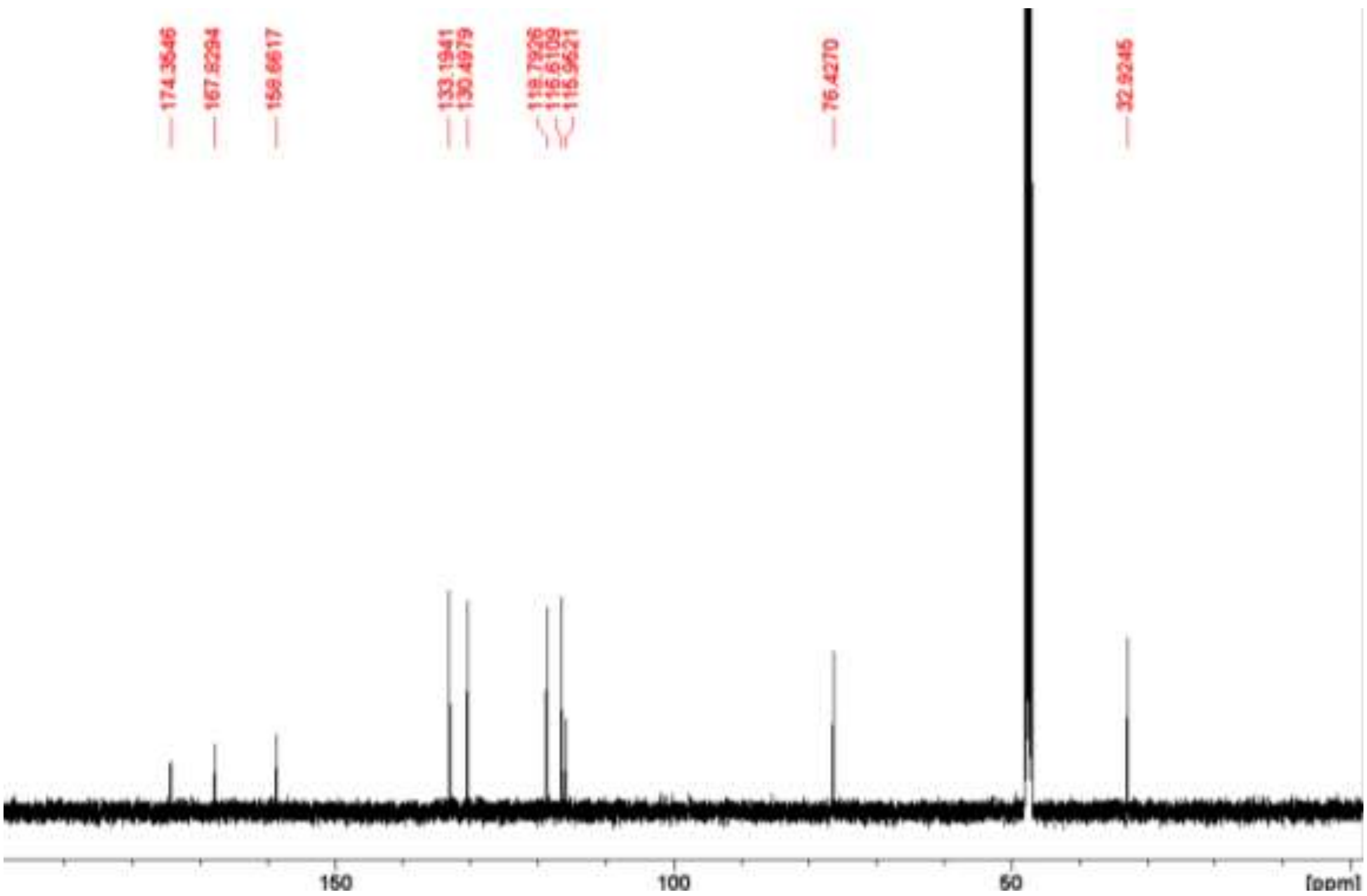

Figure S2. ^{13}C NMR spectrum of compound **L2** measured in CD_3OD.

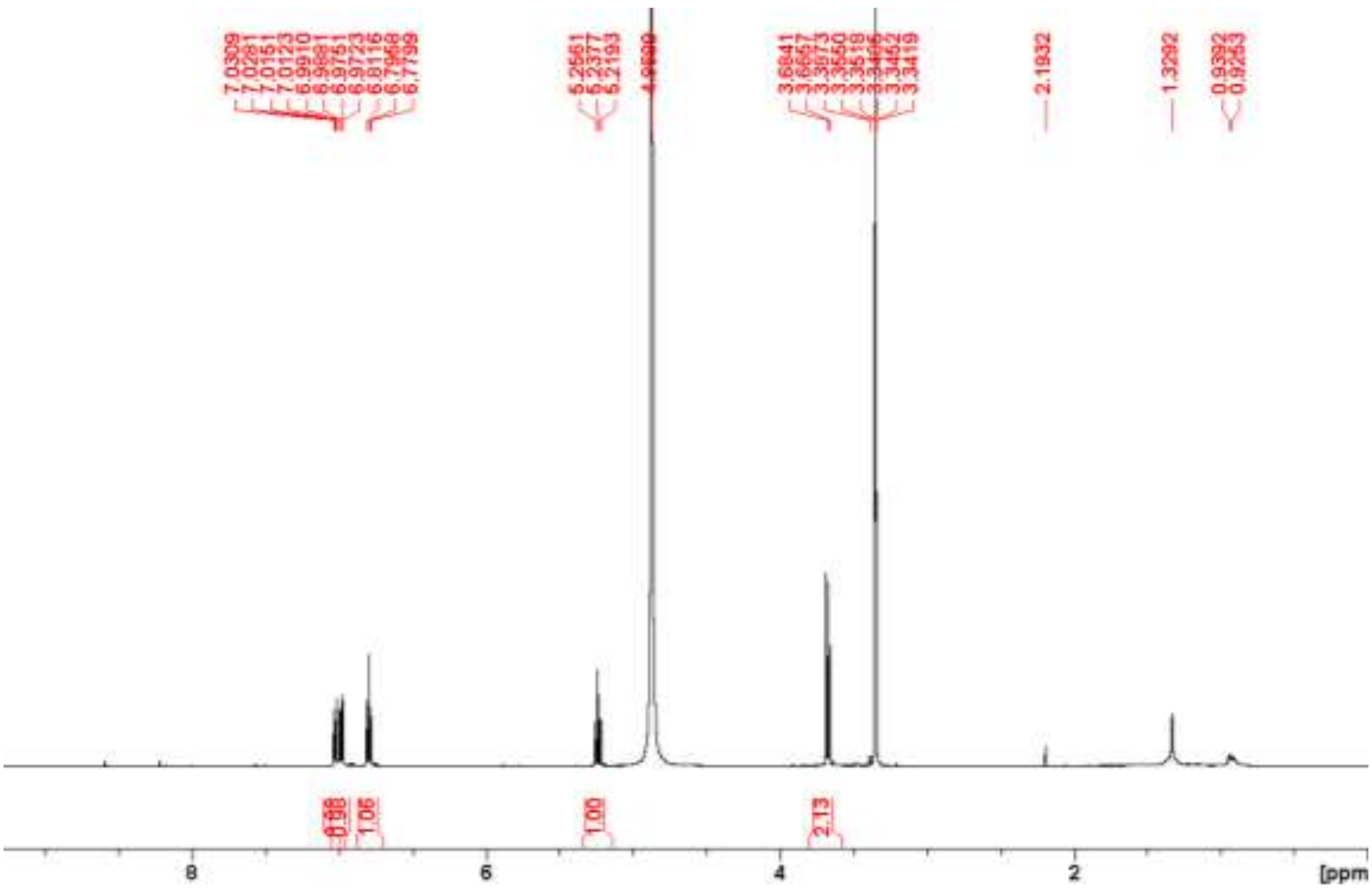

Figure S3. ^{1}H NMR spectrum of compound **L4** measured in CD_3OD

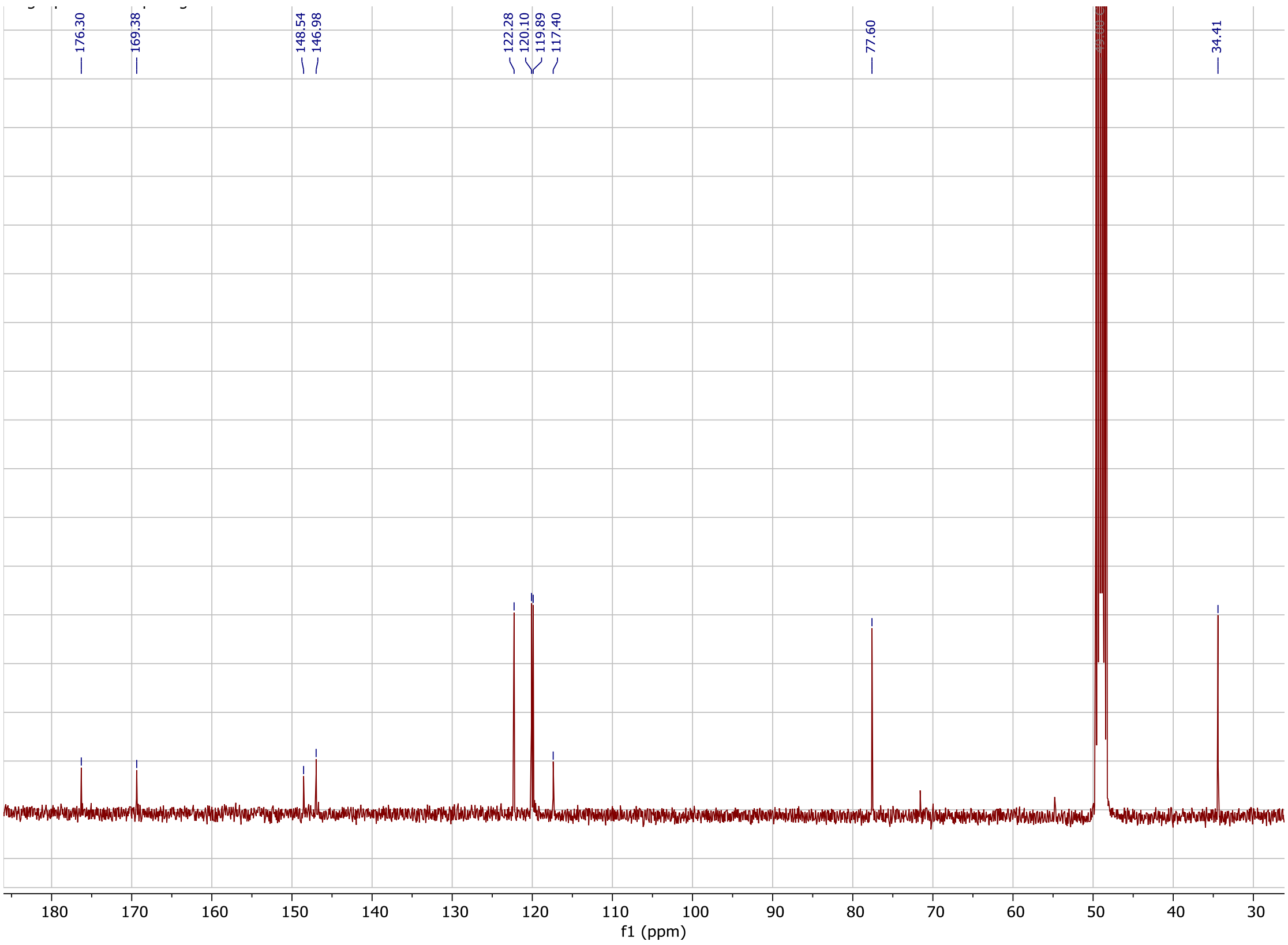

Figure S4. ^{13}C NMR spectrum of compound **L4** measured in CD_3OD.

2. UV-Vis spectra of L1–L4 and Ang

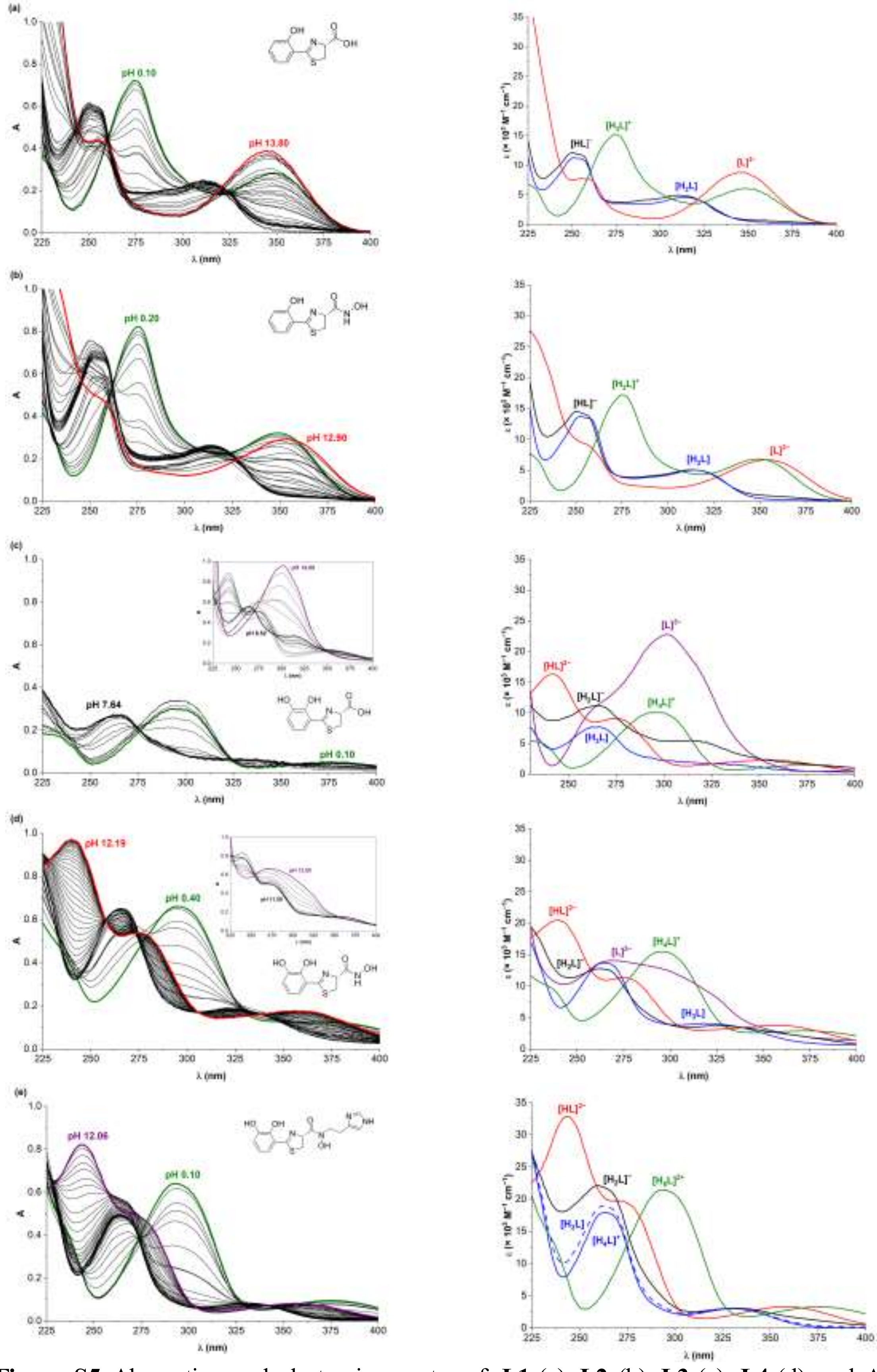

Figure S5. Absorption and electronic spectra of: **L1** (a), **L2** (b), **L3** (c), **L4** (d), and **Ang** (e). C_L range of 3.5–5 × 10^{-5} M, MeOH/H_2O mixture (80:20 w/w), I = 0.1 M $NaClO_4$, T = 25 °C.

Table S1. Electronic absorption spectral data for **L1–L4** and **Ang** in solution[a].

	**L1**		**L2**		**L3**		**L4**		**Ang**	
	λ_{max} [nm]	ε [× 10^3 M^{-1} cm^{-1}]	λ_{max} [nm]	ε [× 10^3 M^{-1} cm^{-1}]	λ_{max} [nm]	ε [× 10^3 M^{-1} cm^{-1}]	λ_{max} [nm]	ε [× 10^3 M^{-1} cm^{-1}]	λ_{max} [nm]	ε [× 10^3 M^{-1} cm^{-1}]
[H_5L]									376	3.3
									294	21.4
									239^{sh}	11.5
[H_4L]					379	1.5	378	2.9	331	3.0
					297	10.1	296	15.5	264	17.9
							236^{sh}	9.7		
[H_3L]	350	6.0	351	6.8	330^{sh}	1.7	319	4.1	331	3.1
	275	15.2	276	17.2	265	7.7	265	13.7	263	18.9
[H_2L]	315	4.6	315	5.0	316	5.5	325	3.7	332	3.1
	254	11.2	257	13.5	265	11.1	263	12.7	260	22.1
	251	11.3	252	13.7						
[HL]	312	4.9	316	5.0	356	2.3	360	3.7	358	3.3
	254	11.8	255	14.1	276	9.0	276	11.3	272	19.7
	251	12.2	251	14.5	242	16.3	240	20.5	244	32.9
[L]	348	8.8	355	6.7	302	22.8				
	258	7.7	256^{sh}	9.2						

[a] Solvent: MeOH/H_2O mixture (80:20 w/w), I = 0.1 M $NaClO_4$, T = 25 °C. Experimental errors λ_{max} = ± 2 nm, ε = ± 5%. Charges are omitted for clarity.

3. Mass spectra of Fe(III) complexes with L1–L4 and Ang

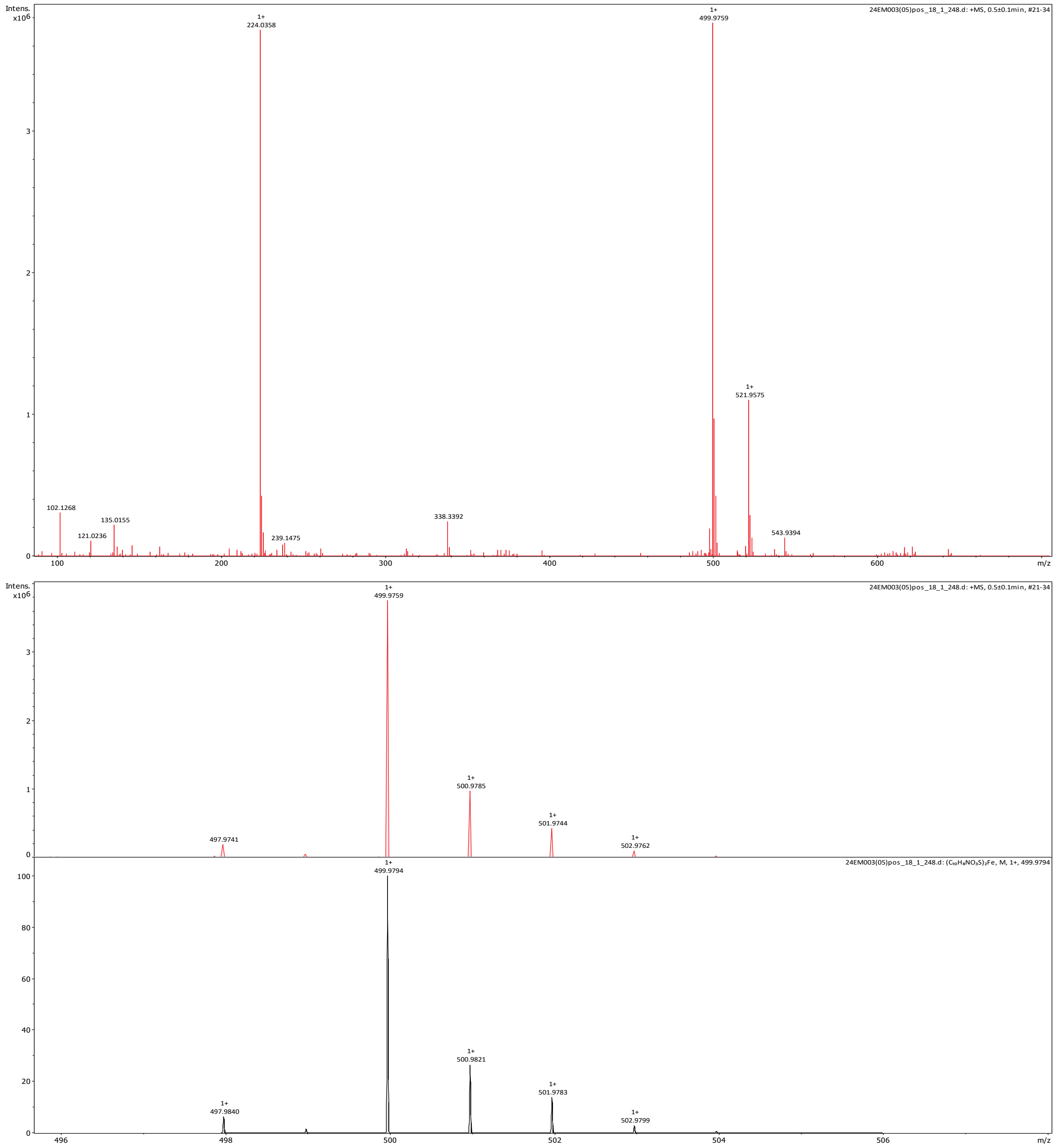

Figure S6. ESI-MS spectrum of the Fe(III)-**L1** complex with a metal-to-ligand ratio of 1:2. Samples were prepared in a MeOH/H_2O solution (80:20 w/w). The final sample concentration was 0.1 mM. The full spectra and expanded experimental (top) and simulated (bottom) spectra of $\{2[HL]^{-}+Fe^{3+}\}^{+}$ (*m/z* = 499.9759) complex.

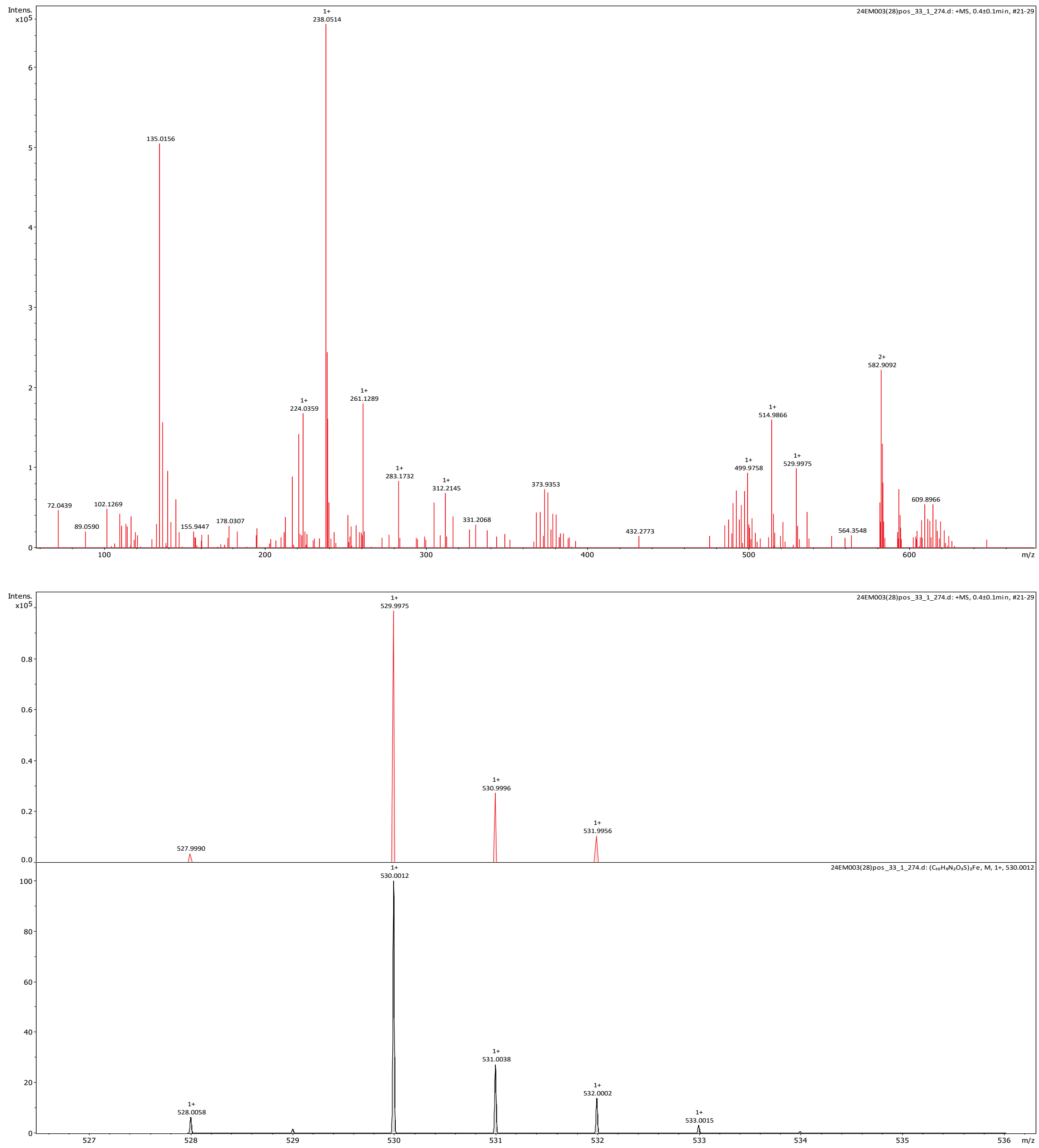

Figure S7. ESI-MS spectrum of the Fe(III)-**L2** complex with a metal-to-ligand ratio of 1:2. Samples were prepared in a MeOH/H_2O solution (80:20 w/w). The final sample concentration was 0.1 mM. The full spectra and expanded experimental (top) and simulated (bottom) spectra of $\{2[HL]^{-}+Fe^{3+}\}^{+}$ (*m/z* = 529.9975) complex.

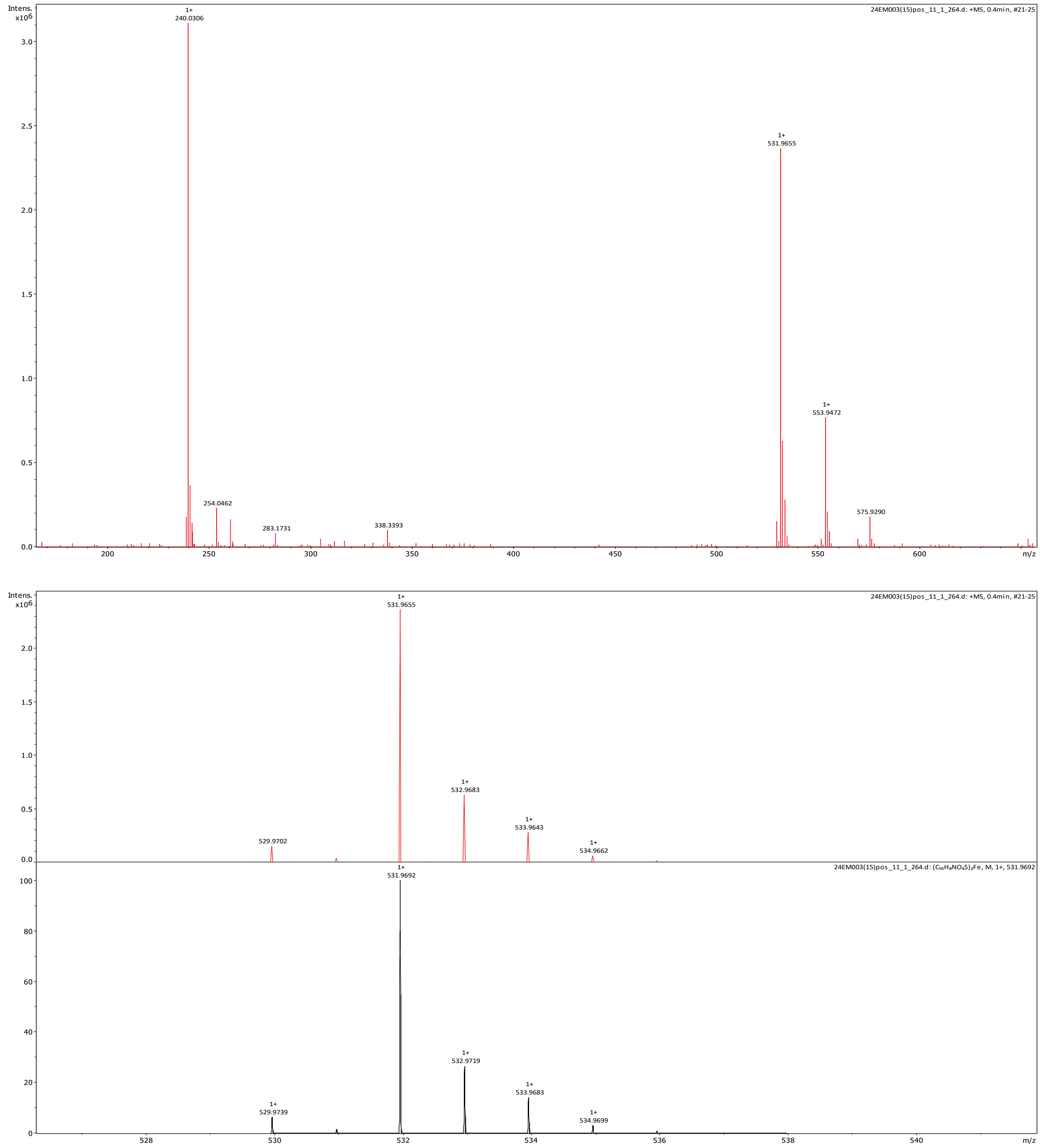

Figure S8. ESI-MS spectrum of the Fe(III)-**L3** complex with a metal-to-ligand ratio of 1:3. Samples were prepared in a MeOH/H_2O solution (80:20 w/w). The final sample concentration was 0.1 mM. The full spectra and expanded experimental (top) and simulated (bottom) spectra of $\{2[H_2L]^{-}+Fe^{3+}\}^{+}$ (*m/z* = 531.9655) complex.

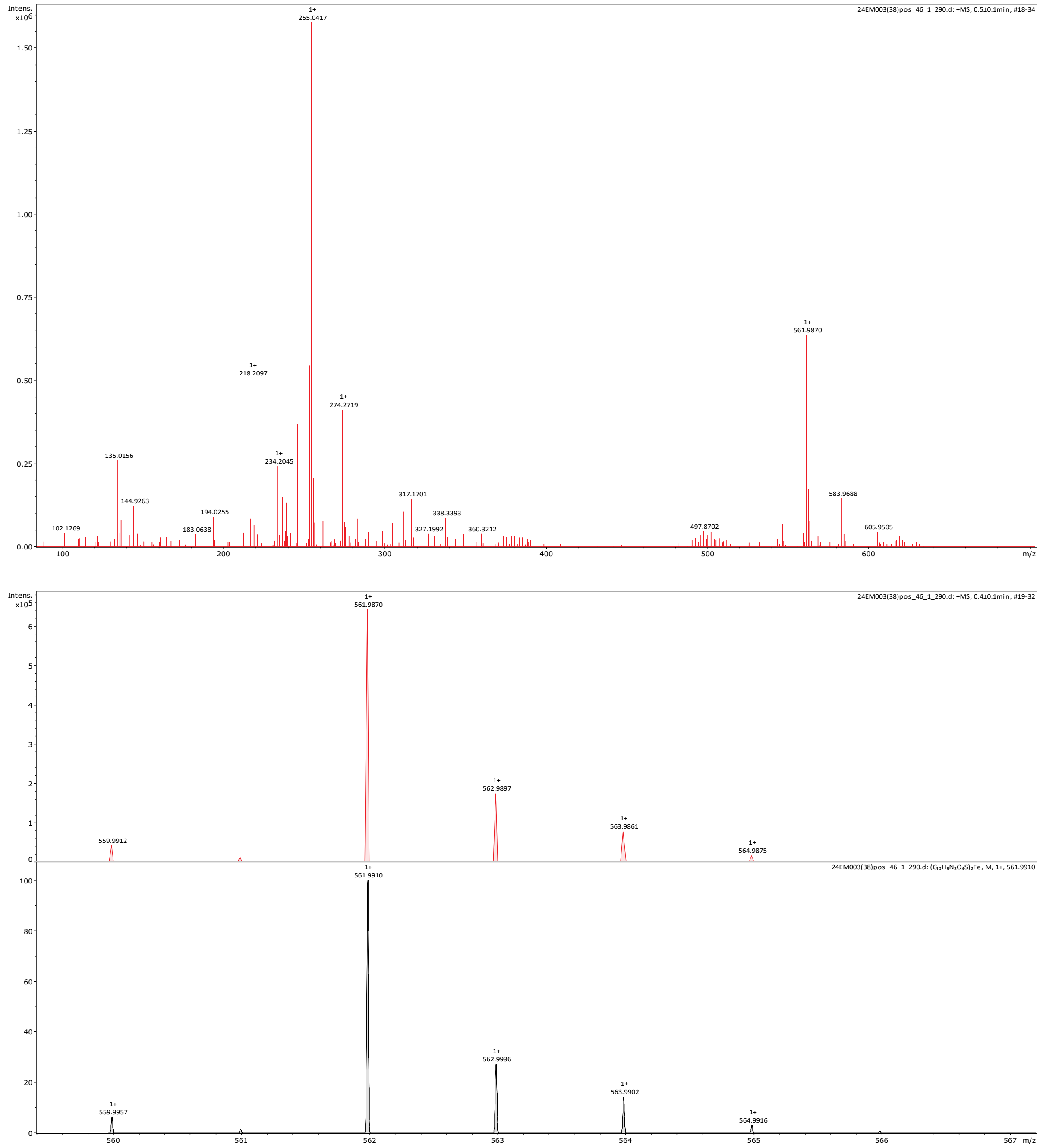

Figure S9. ESI-MS spectrum of the Fe(III)-**L4** complex with a metal-to-ligand ratio of 1:3. Samples were prepared in a MeOH/H_2O solution (80:20 w/w). The final sample concentration was 0.1 mM. The full spectra and expanded experimental (top) and simulated (bottom) spectra of $\{2[H_2L]^{-}+Fe^{3+}\}^{+}$ (m/z = 561.9870) complex.

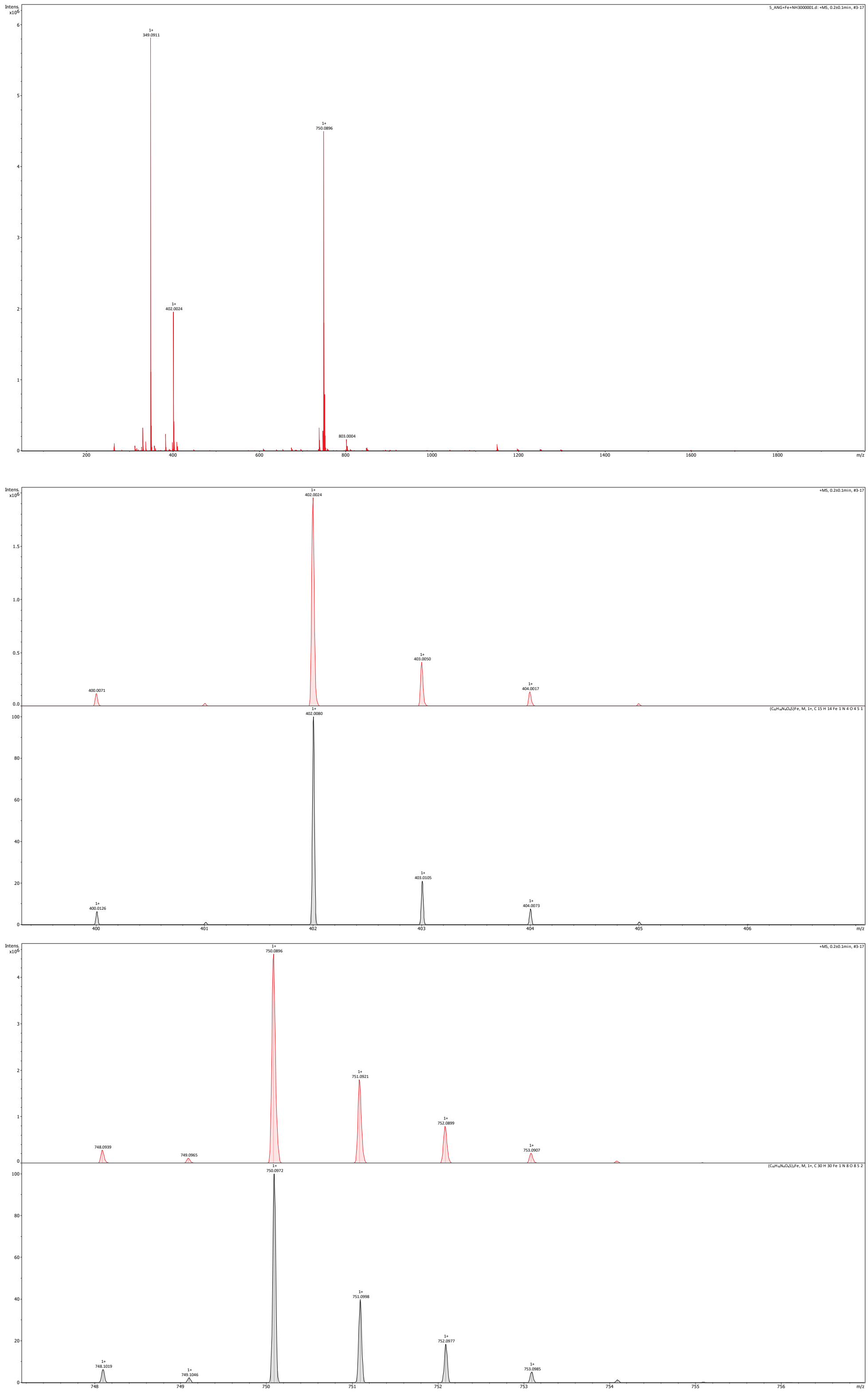

Figure S10. ESI-MS spectrum of the Fe(III)-**Ang** complexes with a metal-to-ligand ratio of 1:2. Samples were prepared in a MeOH/H_2O solution (80:20 w/w). The final sample concentration was 0.1 mM. The full spectra and expanded experimental (top) and simulated (bottom) spectra of $\{[HL]^{2-}+Fe^{3+}\}^{+}$ (m/z = 402.0030) and $\{2[H_2L]^{-}+Fe^{3+}\}^{+}$ (m/z = 750.0909) complexes.

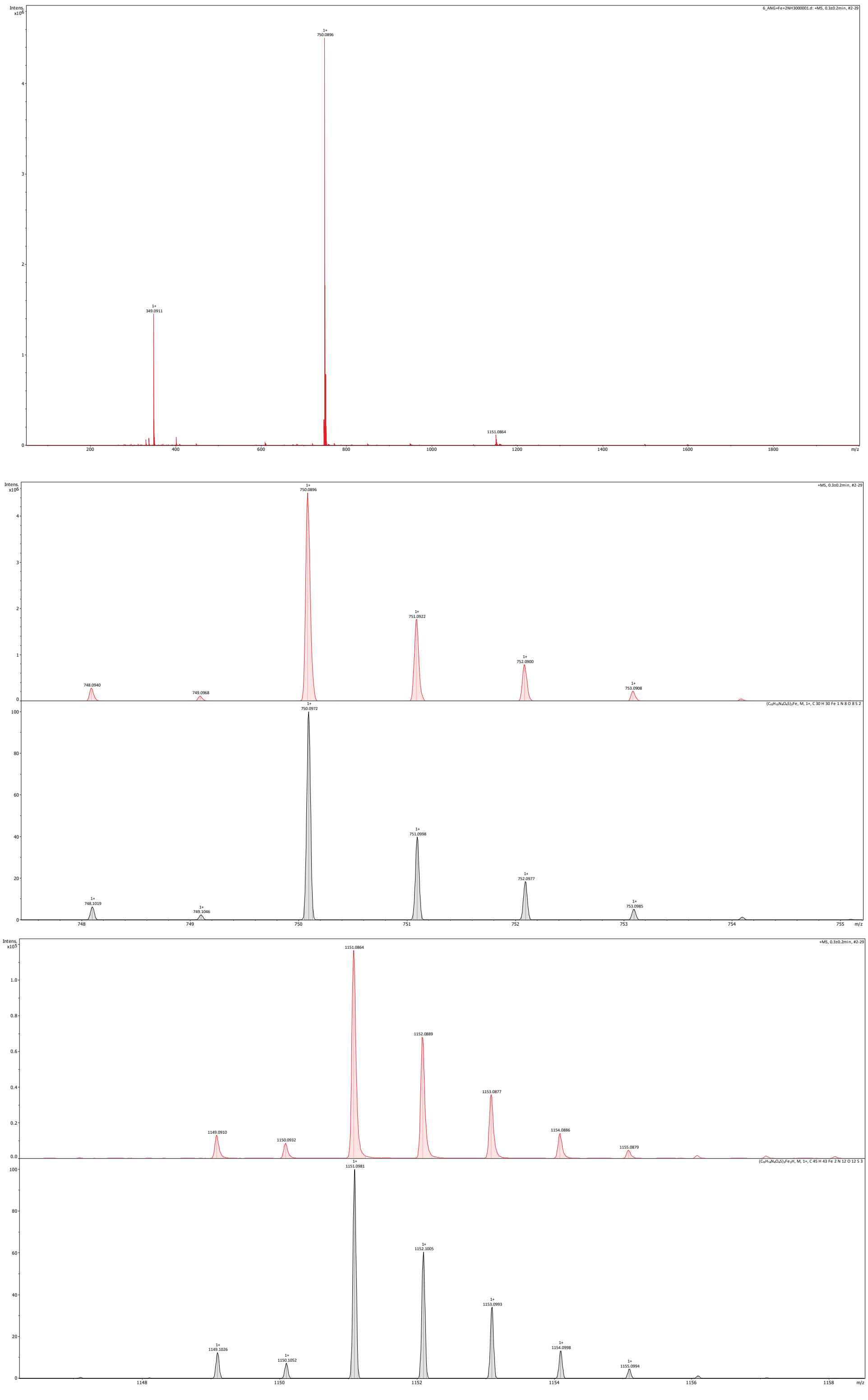

Figure S11. ESI-MS spectrum of the Fe(III)-**Ang** complexes with a metal-to-ligand ratio of 1:2 after pH adjustment with $NH_{3(aq)}$. Samples were prepared in a MeOH/H_2O solution (80:20 w/w). The final sample concentration was 0.1 mM. The full spectra and expanded experimental (top) and simulated (bottom) spectra of $\{2[H_2L]^{-}+Fe^{3+}\}^{+}$ (m/z = 750.0909) and $\{2[HL]^{2-}+[H_2L]^{-}+2Fe^{3+}\}^{+}$ (m/z = 1151.0864) complexes.

4. Distribution Plots of Fe(III) complexes with L1–L4

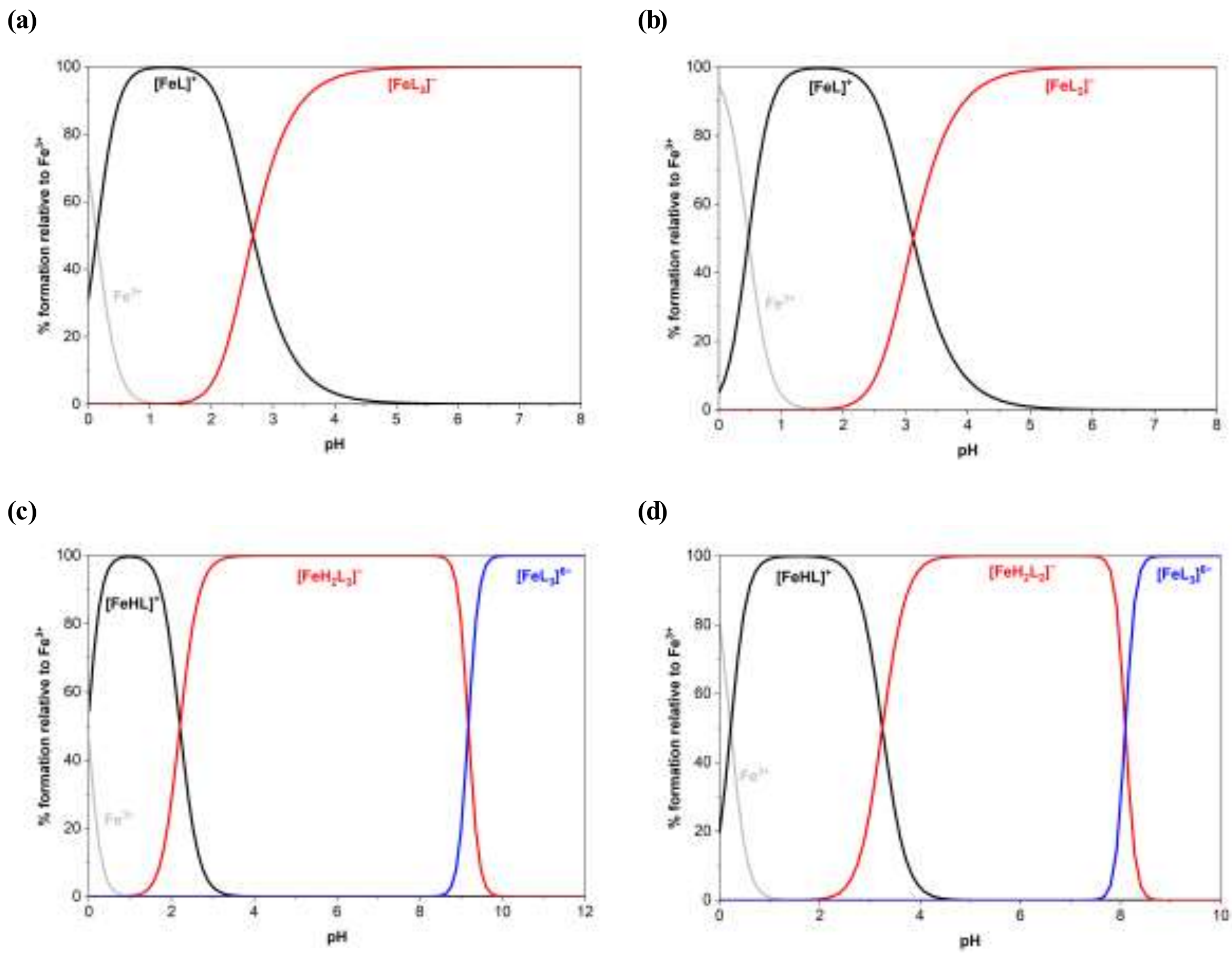

Figure S12. Distribution plots of the **L1**–**L4** ferric complexes as a function of pH. The color coding of the species corresponds to that used in Figure 3; solvent: MeOH/H_2O mixture (80:20 w/w), I = 0.1 M $NaClO_4$, T = 25 °C. For **L1** (a) and **L2** (b): $[L]_{tot} = 2 \times 10^{-4}$ M; $[Fe(III)]_{tot}/[L]_{tot}$ = 0.5; for **L3** (c) and **L4** (d): $[L]_{tot} = 4 \times 10^{-4}$ M; $[Fe(III)]_{tot}/[L]_{tot}$ = 0.25.
